# Impact and cost-effectiveness of interventions to eliminate hepatitis C virus among people who inject drugs in Haiphong, Vietnam

**DOI:** 10.1016/j.drugpo.2025.104898

**Published:** 2025-06-21

**Authors:** Adam Trickey, Josephine G. Walker, Pham Minh Khue, Tran Thi Hong, Nguyen Thanh Binh, Catherine Quillet, Roselyne Vallo, Sandra Bivegete, Khuat Thi Hai Oanh, Hannah Fraser, Duong Thi Huong, Todd Pollack, Vo Thi Tuyet Nhung, Don Des Jarlais, Vu Hai Vinh, Nicolas Nagot, Didier Laureillard, Jack Stone, Peter Vickerman

**Affiliations:** a Population Health Sciences, University of Bristol, UK; b Faculty of Public Health, Hai Phong University of Medicine and Pharmacy, Hai Phong, Vietnam; c Pathogenesis and control of chronic and emergent infections, University of Montpellier, INSERM, Etablissement Français du Sang, University of Antilles, Montpellier, France; d Supporting Community Development Initiatives, Hanoi, Vietnam; e Department of Medicine, Beth Israel Deaconess Medical Center, United States; f Harvard Medical School, Boston, USA; g The Partnership for Health Advancement in Vietnam, Beth Israel Deaconess Medical Center, Ho Chi Minh City, Vietnam; h New-York University, NY, USA; i Infectious and Tropical Diseases Department, Viet Tiep Hospital, Hai Phong, Vietnam; j Department of Infectious Diseases, Caremeau University Hospital, Nîmes, France

**Keywords:** Injecting drug use, DAA, Liver

## Abstract

**Background:**

In Haiphong, Vietnam, most hepatitis C virus (HCV) infections occur among people who inject drugs (PWID). As part of multiple respondent-driven sampling (RDS) surveys among PWID in Haiphong, an intervention (DRIVE-C) provided HCV testing and treatment in 2019. Centres providing opiate agonist treatment (OAT) or antiretroviral therapy (ART) also provided HCV testing and linkage-to-treatment in 2021/22. We modelled the impact and cost-effectiveness of HCV testing and treatment for PWID in Haiphong.

**Methods:**

An HCV transmission model among PWID and former injectors was calibrated in a Bayesian framework using data from Haiphong. A status quo (SQ) scenario modelled past interventions, with no future HCV treatment. A future intervention scenario modelled the impact of providing HCV testing and linkage-to-treatment in OAT and ART centres, and annual RDS survey interventions over 2025–2030, each testing 1400 PWID. We estimated the incremental cost-effectiveness ratio (ICER) per disability adjusted life-year (DALY) averted for the future scenario compared to SQ over 2025–2054 (3 % annual discount rate).

**Results:**

For the SQ scenario, HCV incidence decreased from 8.1 (95 % credibility interval 5.1–13.6) per 100 person-years (/100pyrs) in 2015 to 5.3/100pyrs (3.0–9.6) in 2023 and increases to 6.2/100pyrs (3.5–10.7) in 2030. In the future intervention scenario, incidence decreases to 2.7/100pyrs (1.0–6.4) by 2030. The mean ICER is €884/DALY averted; cost-effective at a willingness-to-pay threshold of €2334 (57 % of Vietnam’s 2023 GDP per capita).

**Conclusions:**

Using RDS surveys and other care settings to scale-up HCV-testing and treatment are cost-effective strategies to reduce HCV incidence among PWID in Vietnam.

## Glossary

HCV: Hepatitis C virus
PWID: People who inject drugs
DAAs: Direct acting antiviral treatments
WHO: World Health Organization
LMICs: Low- and middle-income countries
ART: Antiretroviral therapy
NSP: Needle and syringe programmes
OAT: Opiate agonist treatment
RDS: Respondent-driven sampling
DRIVE: DRug use & Infections in ViEtnam
RNA: Ribonucleic acid
SVR: Sustained virological response
VAAC-GF: Vietnam Administration for HIV/AIDS Control and the Global Fund
Ab: Antibody
SQ: Status quo
ICER: Incremental cost-effectiveness ratio
DALYs: Disability adjusted life years
WTP: Willingness-to-pay
EVPPI: Expected value of partial perfect information analysis

## Introduction

Hepatitis C virus (HCV) is a bloodborne virus that causes significant liver disease resulting in approximately 290,000 deaths each year (World Health Organization, 2023). In Southeast Asia, including Vietnam, it is projected that most incident cases of HCV are among people who inject drugs (PWID) (Trickey et al., 2019). In 2014, highly effective direct acting antiviral treatments (DAAs) for HCV became available (Falade-Nwulia et al., 2017) making it an easily curable infection. This led to the World Health Organization (WHO) setting targets to eliminate HCV as a public health concern by 2030 (World Health Organization, 2016). New guidance from WHO defines HCV elimination among PWID as reaching an incidence rate of 2 infections per 100 person years (Artenie et al., 2022). The availability of low-cost generic DAAs makes this goal a possibility for low- and middle-income countries (LMICs) such as Vietnam (Abozeid et al., 2018).

In 2016, it was estimated that there were 10,000 people with a history of injecting drug use in Haiphong, Vietnam, including 5000 people currently injecting drugs (Des Jarlais et al., 2018) in a city of around 2 million residents, with around 60 % of these PWID having chronic HCV (Nagot et al., 2023). HIV prevalence is also high among PWID in Haiphong (~26 % in 2019 (Des Jarlais et al., 2020)), but HIV incidence is now low due to a high coverage of antiretroviral therapy (ART) (Cohen et al., 2016) among PWID living with HIV, as well as a scale-up of needle and syringe programmes (NSP) since 2005 and opiate agonist treatment (OAT) since 2008 (Moles et al., 2020). NSPs reduce the sharing of used needles and OAT reduces injecting frequency, with both shown to reduce HIV and HCV incidence (Platt et al., 2017; McNaughton, Stone, & Oo, 2023). NSP and OAT will likely have reduced the incidence of HCV among PWID in Haiphong, but the incidence is still high: 19.4 seroconversions per 100 person-years reported in 2017 for active injectors (Moles et al., 2020). So, other interventions are also needed to further reduce incidence.

The availability of HCV testing for PWID in Haiphong was very limited prior to 2014, and affordable HCV treatment was unavailable, which was also the case elsewhere in Vietnam (Des Jarlais et al., 2020; Des Jarlais et al., 2016; Nagot et al., 2023). However, Haiphong has often led Vietnam in the use of evidence-based treatment for substance use and infectious diseases among PWID, possibly due to Haiphong’s high prevalence of HIV (Des Jarlais et al., 2016). Since 2014, because of this high HIV prevalence there have been several initiatives to understand the HIV epidemiology among injectors in Haiphong as well as to increase access to HCV testing and treatment to reduce levels of HCV prevalence and transmission. The DRIVE-IN pilot study in 2014 was a serosurvey that combined respondent-driven sampling (RDS) with peer support groups to test PWID for HIV and HCV antibodies and link them to HIV services and OAT (Des Jarlais et al., 2016). Following this pilot, four similar, but larger RDS surveys (RDS1-4) were undertaken between 2016 and 2019 that included testing for HCV antibodies (Des Jarlais et al., 2020; Des Jarlais et al., 2021). People in these surveys were also recruited to the DRug use & Infections in ViEtnam (DRIVE) cohort and were followed up every 6 months, where possible (Nagot et al., 2023). In the 2018 survey (RDS3), the DRIVE-C project provided testing for HCV ribonucleic acid (RNA) to identify people with chronic HCV infection (Nagot et al., 2023). DRIVE-C also offered RNA testing to people in the existing DRIVE cohort and provided treatment for all PWID diagnosed with chronic HCV (Nagot et al., 2023). The DRIVE-C intervention treated 979 PWID, with a sustained virological response (SVR; effective cure) of 91.7 % (Nagot et al., 2023). From February 2021 to June 2022, a partnership between the Vietnam Administration for HIV/AIDS Control and the Global Fund (VAAC-GF) project also tested and treated people with HCV across Vietnam (Vietnam Administration for HIV/AIDS Control, 2023). For Haiphong, the VAAC-GF project treated 640 people with HCV attending ART centres and 191 attending OAT centres; 99 % had acquired HCV through injecting drug use. Since these interventions, there has been little testing or treatment for HCV in Haiphong.

These interventions show that it is feasible to test and treat PWID for HCV in Haiphong. There is potential for scaling up testing and treatment for HCV through further RDS surveys and providing additional testing and treatment in OAT and ART centres. We used mathematical modelling to evaluate the impact and cost-effectiveness of previous and future interventions to increase testing and treatment for HCV among PWID in Haiphong, Vietnam.

## Methods

### Model summary

**Fig. 1:**
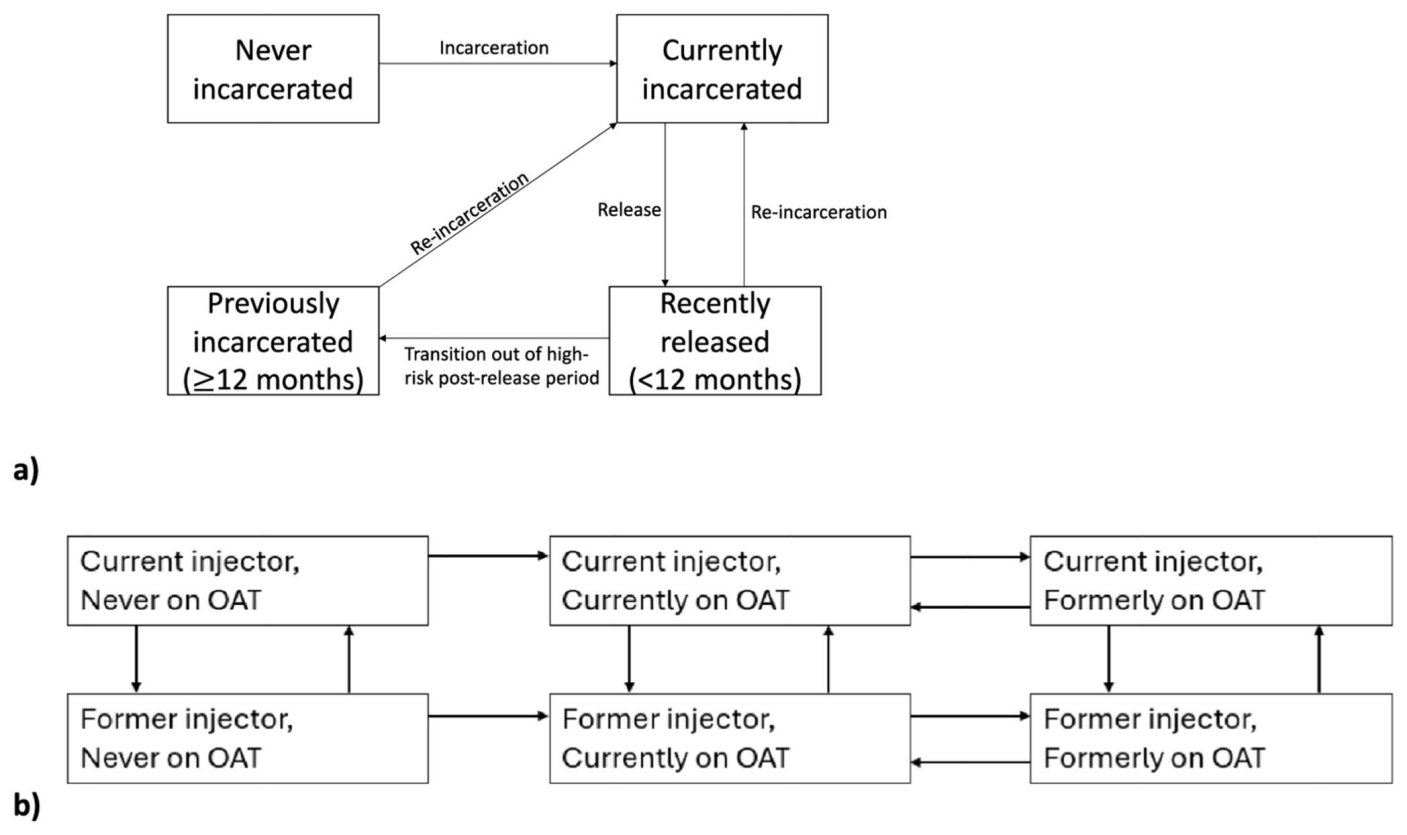
a-b: Model schematics for (a) incarceration status and (b) injecting and opioid agonist treatment status.

**Fig. 2:**
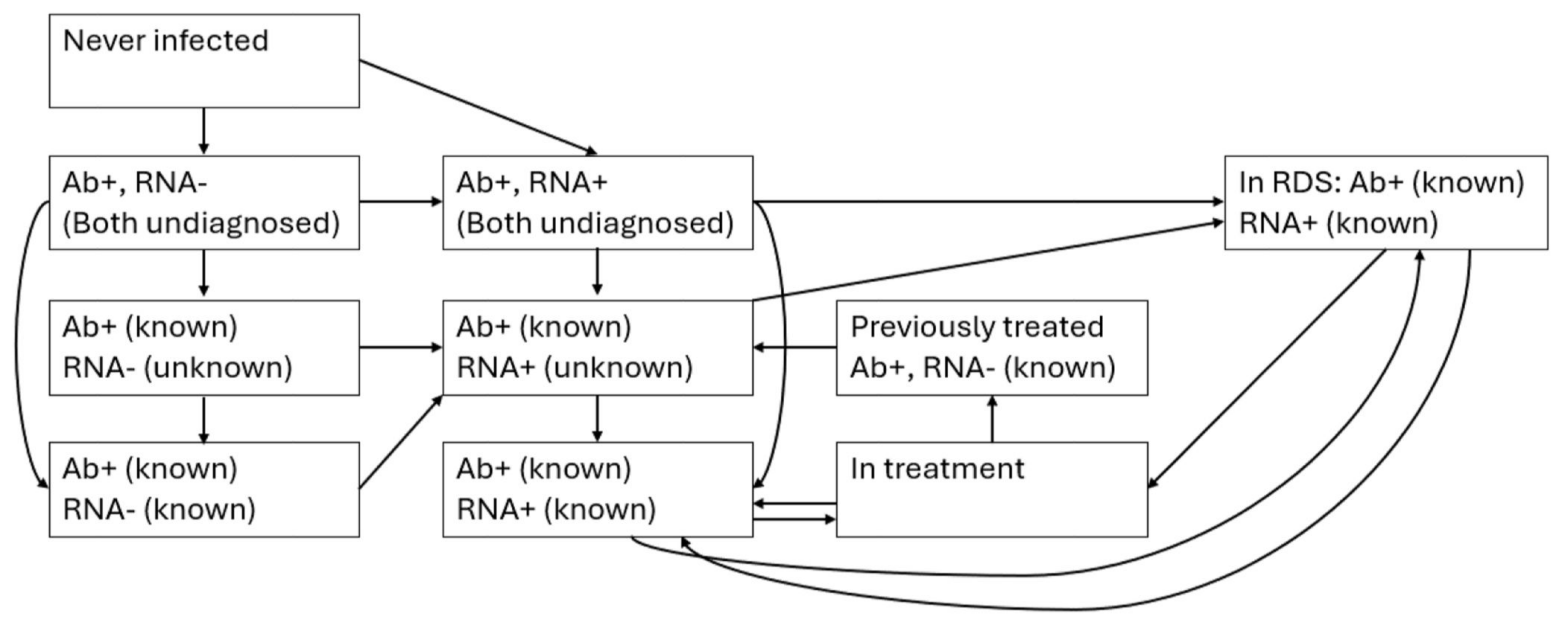
Hepatitis C virus infection, diagnosis and treatment status schematic. Ab: Antibody. RNA: Ribonucleic acid. RDS: Respondent-driven sampling survey.

We developed a dynamic, deterministic model of HCV transmission among current and former PWID in Haiphong, Vietnam. A detailed description of how people enter and move through the model is given in the supplementary materials. The model is stratified by: low/high-risk injecting status (low-risk, high-risk without HIV, high-risk with HIV), age (16–39, ≥40), incarceration status (Fig. 1a), current injecting and OAT status (Fig. 1b), HCV infection, diagnosis and treatment status (Fig. 2), and disease progression status (metavir states F0-F4, decompensated cirrhosis, hepatocellular carcinoma). Model schematics are in Figs. 1, 2 and supplementary figures 1–4. The high-risk injecting category, which is stratified by HIV status, is used to capture the effect of HIV infection status being strongly associated with HCV acquisition (based on HCV incidence data from the DRIVE study) and mortality, and to enable us to project the impact of interventions in ART centres. However, we do not model HIV transmission because incidence is low among PWID in Haiphong due to high ART coverage (plus NSP and OAT); no seroconversions were observed in the DRIVE study over 206 person-years of follow-up (Moles et al., 2020). We do not model differences in HCV progression by HIV status due to high coverage levels of ART, which ameliorates the effects of HIV on HCV disease progression (Thein et al., 2008), but we do model interventions that preferentially screen people attending ART centres.

The model assumes that HCV transmission only occurs between active PWID either in prison or the community. The HCV force of infection is dependent upon the HCV chronic prevalence among active PWID in each setting (prison or community) and this relative risk is reduced for PWID on OAT or aged ≥40 (based on HCV incidence data from the DRIVE study), but is heightened among those who are high-risk or have been recently released from prison (Stone et al., 2018). These relative risks are multiplicative. We also allow the HCV force of infection to differ for those currently incarcerated.

There are 10 HCV infection, diagnosis, and treatment categories in the model as shown in Fig. 2 and described further in the supplement. People start off as never infected and then, when infected, move to either the undiagnosed and uninfected (antibody (Ab)+ and ribonucleic acid (RNA)-) or undiagnosed and infected (Ab+ and RNA+) categories, depending on the proportion that spontaneous clear their infection. The model then accounts for Ab and RNA testing by incorporating categories relating to these diagnosis states. People in the diagnosed infection (Ab+ known, RNA+ known) category can initiate treatment. Successfully treated individuals move to the previously treated and uninfected (Ab+ and RNA-known) category, from where they can be reinfected and move back into an undiagnosed infection category, but with known Ab+.

Testing and treatment mainly occur through historical and future interventions (through RDS and at OAT/ART centres), with a low background level of Ab testing present from 2010 onwards (fit to proportion ever HCV tested in RDS1 survey). There is a separate category for people who are diagnosed HCV RNA+ through RDS surveys, as only people who have been diagnosed through RDS surveys are treated through an RDS survey. If someone was diagnosed through another mechanism (e.g. in an OAT centre), they could still be retested through the RDS, as everyone participating in the RDS is tested. When people are incarcerated, they cease contact with OAT and RDS surveys.

### Model parameterisation, calibration, and validation

Most data used to parameterise and calibrate the model were taken from the 4 RDS surveys of current injectors done in Haiphong over 2016–2019 [*N* = 1383, 1451, 1444, and 1268, respectively] (Des Jarlais et al., 2021), the DRIVE-IN survey from 2014 [*N* = 603] (Des Jarlais et al., 2016) and the DRIVE-C survey from 2019 [*N* = 1425] (Nagot et al., 2023) (Table 1). Data from the DRIVE cohort, recruited through the RDS surveys, were also used. Data on the scale-up of individuals attending OAT clinics in Haiphong (from 0 to 3898 over 2008–2019) were used to determine the scale-up of OAT in the model. Increased linkage to OAT was assumed when the RDS surveys were occurring, as this was an aim of the DRIVE program (Des Jarlais et al., 2021). Data on the number of people tested by each survey were also included in the model. All the surveys included HCV antibody testing, whilst DRIVE-C provided RNA testing through RDS3 and the DRIVE cohort, with HCV treatment provided anyone that was diagnosed (Des Jarlais et al., 2021; Nagot et al., 2023). Additionally, the VAAC-GF project tested and treated people with HCV in OAT and ART centres over 2021–2022; 99 % of which reported injecting drug use as a risk factor. Numbers tested and diagnosed are given below. The percentage achieving SVR in DRIVE-C was 91.7 % (629/686) which was assumed for all treatments. Following successful treatment, we assumed a reduced risk of re-infection (Risk ratio=0.46) because the reinfection rate following treatment (4.1 per 100 person-years (Nagot et al., 2023)) in Haiphong is lower than the primary incidence rate (8.9 per 100 person-years calculated from the DRIVE cohort). Details on the model parameters are in the supplementary materials.

We calibrated the model using Approximate Bayesian computation sequential Monte Carlo (ABC SMC) scheme to the data listed in Table 1. This method was chosen due its computational efficiency (Toni et al., 2009). The algorithm begins with 1000 parameter sets sampled from prior distributions (supplementary table 1), which are then resampled and perturbed in an iterative manner to better fit the data; for 30 iterations. The resulting 1000 model fits were used to estimate the median and 95 % credibility intervals (95 %CrI; 2.5th-97.5th percentile range) for all model projections. We validated the model against various data measures from the DRIVE studies (Table 1). A detailed description of model parameterisation, calibration, and validation is given in the supplementary materials.

### Modelling impact of existing and future interventions

The modelled SQ scenario (scenario 1) as described above contains all existing HCV testing and treatment interventions (Table 2), with no further HCV testing and treatment after 2022. A testing rate was estimated for each RDS survey to give the number tested in each survey, while antibody and RNA testing rates were estimated for DRIVE-C so that the correct number were tested over 0.25 of a year. We calibrated a DRIVE-C linkage-to-treatment rate to give the number of people treated (979) over 1.25 years. The testing rates for VAAC-GF were estimated through calibration to give the number of PWID diagnosed with HCV in ART and OAT centres over 1.25 years in 2021–2022, and similarly for the numbers treated in ART and OAT centres.

When the RDS surveys are taking place we allow for a different rate of recruitment to OAT, as this was an objective of the RDS surveys. This was done by assuming the rates of (re)starting OAT during the RDS surveys are multiplied by a relative risk (0–1) during the periods without RDS surveys, which are estimated through the calibration process.

The status quo (SQ) scenario was compared to a full counterfactual scenario (scenario 0a) where no current or former injectors were diagnosed or treated in the surveys, linkage to OAT was not improved whilst the RDS surveys were occurring, and no testing and treatment occurred through OAT and ART centres. A partial counterfactual scenario (0b) was also evaluated to determine the cost-effectiveness of the DRIVE-C intervention. The SQ scenario 1 was also compared with various scenarios that included additional future interventions from 2025 to 2030 (Table 2). These either continued the VAAC-GF intervention at OAT and ART centres or undertook additional RDS surveys with HCV treatment.

For all scenarios that include HCV testing and treatment in ART and OAT centres, we assumed people would be tested and linked to HCV treatment at the same yearly rate as was estimated for the VAAC-GF intervention. For all scenarios including annual RDS surveys, each RDS survey was assumed to test 1400 people (antibody tested and RNA tested if Ab+) in the first quarter of each year from 2025 to 2030, with diagnosed individuals being treated as in DRIVE-C. Current and former injectors in the community, at any disease progression stage, were/are treated in the OAT/ART interventions, with just current injectors being treated in the RDS interventions. During each RDS survey, recruitment to OAT was higher, as estimated for previous RDS.

### Cost-effectiveness of existing and future interventions

Unit costs used in the model were estimated using detailed micro-costing methodology (Supplementary Materials, supplementary table 3). We conducted a retrospective costing analysis, with a custom costing tool developed through site observations, discussion with project staff, and record review in February 2020, and expenditure data from DRIVE-C and RDS screening from 2018 to 2019 included in the analysis. The cost calculation allocates indirect costs such as non-patient facing staff costs, buildings, annualised equipment costs, and supplies, while direct costs consist of staff time-based event costs, lab costs, drugs, and patient transport. The cost per person for the RDS surveys and GeneXpert were calculated separately from treatment costs.

The incremental cost-effectiveness ratio (ICER) of the SQ scenario 1 was firstly estimated in terms of costs (2023 Euros) per disability adjusted life years (DALYs) averted over a 30-year time horizon (2014–2043) compared with the counterfactual scenario 0a or 0b The ICERs of all future HCV testing and treatment scenarios were then estimated over 30 years (2025–2054) compared with the SQ scenario (scenario 1). Costs and DALY weights were applied as described below, applying a 3 % annual discount to both. The ICER for each intervention was calculated as the mean incremental costs for the 1000 model fits, divided by the mean incremental DALYs. A fully incremental cost-effectiveness analysis was also conducted which involves ordering the intervention scenarios in terms of increasing cost, and based on mean incremental cost and DALYs averted, eliminating any interventions which are dominated (higher cost and fewer benefits) or extendedly dominated (higher ICER and fewer benefits) to determine the most cost-effective intervention scenario. Cost-effectiveness was evaluated using a willingness-to-pay (WTP) threshold of 57 % of Vietnam’s per capita GDP in 2023 (€4095, 57 % is €2334), taken from literature on WTP thresholds specific to Vietnam (International Monetary, Fund; Ochalek et al., 2019). Cost-effectiveness acceptability curves were presented to show how the choice of scenario varies by WTP threshold, accounting for uncertainty in the model runs.

DALYs were calculated using disability weights assigned to liver disease states in the model. Disability weights were taken from the 2019 Global Burden of Disease study (Salomon et al., 2015) (supplementary table 2). For assessing the cost-effectiveness of the SQ scenario 1, we included costs for the RDS interventions (including HCV antibody and RNA testing), HCV RNA and antibody testing in OAT and ART centres, and HCV treatment costs, including staff costs for each. For assessing the cost-effectiveness of each future scenario, we similarly included the costs of the future interventions. Costs were estimated from the perspective of an external investor adding HCV testing and treatment to existing services and/or undertaking RDS surveys, rather than from the government.

In separate sensitivity analyses that altered the assumptions of the SQ scenario 1 and scenario 4 (and the cost-effectiveness analysis comparing the two), we investigated the implications of: including costs for OAT; using disability weights from the 2021 Global Burden of Disease study (Diseases & Injuries, 2024) (supplementary table 4); including healthcare costs for HCV disease estimated from a study in Cambodia (Walker et al., 2020) (supplementary table 5); using a shorter time horizon (to 2044 rather than 2054); and using a 0 % discount rate. To evaluate the relative impact of variation in parameter inputs we conducted an expected value of partial perfect information analysis (EVPPI) (Fenwick et al., 2020). This ranked the relative importance of each parameter to the total expected value of perfect information in terms of its impact on the decision of which intervention is most cost-effective, comparing scenario 4 versus the SQ at the €2334 WTP threshold. Further details about the cost-effectiveness analysis are given in the supplementary materials.

All analyses were carried out in Matlab 2022a, except the EVPPI, which was performed in R using the BCEA package (Baio et al., 2017).

## Results

### Comparison of the status quo model with data

**Fig. 3:**
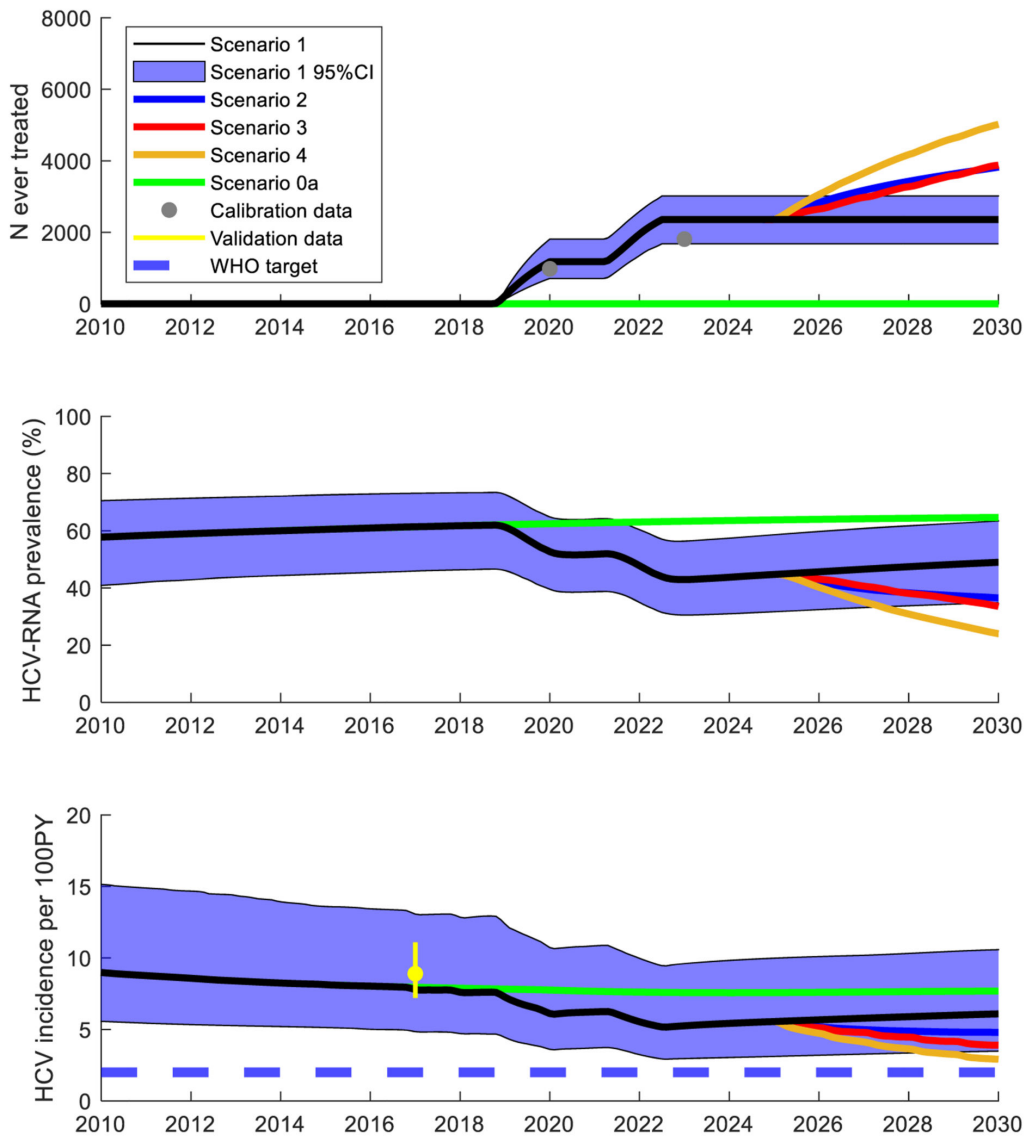
Model projections of the number of PWID and ex-injectors treated for HCV, community chronic prevalence (HCV RNA positive), and community HCV incidence rate. 100PY: 100 person-years

Supplementary figures 5–11 and Fig. 3 show that the SQ model generally agreed well with the calibration data, except for the percentage of active injectors who are aged ≥40 and the number of active PWID, which are both slightly overestimated. The model also agreed well with the validation data not used in the model calibration (green dots in supplementary figures 5–11), including the overall community HCV incidence.

### Impact of existing and future interventions

As of 2023, the model estimated 2355 (95 %CrI: 1679–3017) HCV treatments had been given due to the DRIVE-C and VAAC-GF interventions, compared with 6248 (4489–8149) people who had HCV in 2016, while the number of people on OAT had increased from 3407 in 2014 to 3924 in 2017. Due to these interventions (SQ scenario), the model estimates that the community HCV incidence using a denominator of both active and former injectors decreased from 8.1 (5.1–13.6) per 100 person-years in 2015 to 5.3 (3.0–9.6) in 2023, rising to 6.2 (3.5–10.7) by the end of 2030 with no further interventions, a 25 % (9–37 %) decrease compared to 2015 (Fig. 3 and Table 3; reinfection incidence estimates in supplementary figure 9). In contrast, without any historical HCV testing and treatment interventions since 2014 (Scenario 0a), the estimated community HCV incidence rate per 100 person years in 2020 would be 7.8 (4.8–13.2), 7.6 (4.7–13.0) in 2023 and 7.7 (4.8–13.3) in 2030. Conversely, with just the historical RDS3 and DRIVE-C survey interventions removed (Scenario 0b), the estimated community HCV incidence rate per 100 person years in 2023 is 6.3 (3.7–11.3) and 7.0 (4.1–12.0) in 2030.

Regarding future interventions, administering HCV testing and treatment in ART and OAT centres over 2025–2030 (scenario 2) gives a community HCV incidence rate of 4.8 (2.3–8.9) per 100 person-years in 2030, a 43 % (26–59 %) decrease compared to 2015 (Fig. 3 and Table 2), through administering 1608 (884–2596) additional treatments. Implementing annual RDS surveys (with treatment) over 2025–2030 (scenario 3) produces an HCV incidence rate of 3.7 (1.5–8.1) per 100 person-years by the end of 2030, with 1858 (8814–3209) treatments administered. Combining the annual RDS surveys with HCV testing and treatment at OAT and ART centres (scenario 4) gives an incidence rate of 2.7 (1.0–6.4) per 100 person-years in 2030, a 67 % (46–84 %) decrease compared to 2015. This scenario resulted in 2986 (2035–4362) treatments administered and has a 29 % probability of decreasing the incidence below 2 per 100 person-years. The estimated number of current and former injectors in Haiphong with decompensated cirrhosis and hepatocellular carcinoma (HCC) in 2030 and the number of all-cause deaths occurring in 2030 is given in supplementary table 8. This shows that scenario 4 has around 60 % of the HCC cases in 2030, 33 (11–66), as in the SQ, 55 (22–96).

### Cost-effectiveness of existing and future interventions

Through comparing the SQ (scenario 1) with the full counterfactual (scenarios 0a, all previous testing and treatments removed), we estimate that 1979 DALYs (discounted at 3 % annually) would be averted over 2014–2043 by the testing and treatments undertaken by the RDS surveys, DRIVE-C and VAAC-GF over 2014–2022. Conversely, 886 DALYs would be averted by just the testing and treatments undertaken by DRIVE-C (scenario 0b). The discounted incremental costs related to the RDS, DRIVE-C and VAAC-GF interventions over 2014–2043 was 2516,512 euros, while it was 941,888 euros without the DRIVE-C interventions. This produces an ICER of 1271 Euros/DALY averted for the RDS, DRIVE-C, and VAAC-GF interventions and 1063 Euros/DALY averted for just DRIVE-C (supplementary tables 5 and 6).

Compared to SQ scenario 1, Table 4 shows that all future interventions are cost-effective over 2025–2054 compared to the WTP threshold of €2334 per DALY averted, with 100 % of runs being cost-effective for all scenarios. The intervention that gets closest to the WHO incidence target, combining annual RDS surveys with HCV testing and treatment at OAT and ART centres (Scenario 4), would avert 2815 DALYs over 2025–2054 at an incremental cost of €2488,612, giving an ICER of €884/DALY averted (vs status quo). The fully incremental cost-effectiveness analysis (Table 4) (Paulden, 2020) shows that scenario 2 would be the most cost-effective scenario, with an ICER of €410/DALY averted compared to scenario 1 – averting 2188 DALYs at a cost of €897, 643. Scenario 3 (annual RDS surveys) was dominated by scenario 2 (HCV treatment in ART and OAT centres) as it averted fewer DALYs at a greater cost. The ICER for scenario 4 compared to scenario 2, €2536/DALY, was above the WTP threshold (cost-effectiveness acceptability curve in supplementary figure 12) as it averted more DALYs compared with the status quo (2815 vs 2188), but at a much greater cost (€2488,612 vs €897,643).

In the sensitivity analyses, including the cost of administering OAT in the SQ and scenario 4, the ICER increases but is still cost-effective at 1909 euros/DALY averted compared to scenario 1 (SQ). In the sensitivity analysis using the 2021 Global Burden of Disease disability weights, the ICER for scenario 4 becomes 1069 euros/DALY averted compared to SQ. In the sensitivity analysis where we include healthcare costs for HCV disease, the ICER for scenario 4 becomes 462 euros/DALY averted. Having no discounting on costs and DALYs over time produced an ICER of 565 euros/DALY averted. The EVPPI analysis found that at the WTP threshold of 2334 euros/DALY averted, the rate of leaving incarceration (which interrupts the HCV-related interventions) was the parameter with the largest impact on the decision of which scenario to choose, followed by the starting number of PWID and former injectors. Supplementary figure 13 summarises this information for all 59 parameters.

## Discussion

Previous interventions seeking to implement HCV testing and treatment among PWID in Haiphong are cost-effective and have decreased HCV incidence among PWID by 36 % from 2015 to 2023. Despite this impact, the HCV incidence among PWID is still high (5.3 per 100 person-years in 2023) because around two-thirds of infections in PWID have not been treated. Considerable scale-up of HCV testing and treatment interventions are needed to reduce HCV incidence to low levels among PWID in Haiphong. Indeed, to reduce HCV incidence to close to the WHO target of 2 per 100 person-years requires HCV testing and treatment at OAT and ART centres with six annual RDS surveys over 2025–2030 providing HCV testing and linkage-to-treatment. This intervention involves treating 2986 (2035–4362) individuals and costs €2488,612, averting 2815 DALYs at a cost of €884 per DALY averted, well below the estimated WTP threshold for Vietnam of €2334/DALY. In contrast, if no future treatments were undertaken, the HCV incidence in Haiphong will remain high at 6.2 (3.5–10.7) infections per 100 person-years in 2030.

### Comparison with other literature

To our knowledge, this is the first study to investigate the cost-effectiveness of interventions for HCV among PWID in Vietnam. A previous study modelled the impact, but not cost-effectiveness, of HCV treatment among PWID in Ho Chi Minh City, Vietnam, which, as in our study, found that HCV treatment could reduce infections and deaths among PWID, whilst OAT would reduce HCV incidence (Birger, Le, Kouyos, Grenfell, & Hallett, 2017). Another study modelled HCV treatment as prevention among PWID in Vietnam, which found that treating PWID would avert future infections and would be more effective when combined with OAT (Durier, Nguyen, & White, 2012). Other studies have modelled the HCV epidemic in Vietnam (Blach et al., 2022; Heffernan et al., 2019; Stone et al., 2022; Trickey et al., 2019), but as part of global HCV transmission models. Studies in the broader region have modelled HCV transmission in various populations, mostly focusing on describing the HCV epidemic in their settings (Jia et al., 2019; Wang et al., 2020; Zhang & Zhou, 2012), whilst one study of HCV elimination in Singapore found that targeting HCV treatment to PWID and scaling up OAT would be the most effective strategy to achieve the WHO HCV targets (Chaillon et al., 2021). Studies have investigated the cost-effectiveness of real-life interventions for HCV among PWID in other settings, but few in LMICs (Mafirakureva et al., 2022; Stone et al., 2023), and none in Southeast Asia. One study in Cambodia showed the cost-effectiveness of a simplified real-world treatment pathway for HCV (Walker et al., 2020), but did not focus on PWID. Another cost-effectiveness study of HCV in Bangkok, Thailand, found early treatment would be cost-effective, but this was among men who have sex with men (Shreoshee Mukherjee et al., 2021). A cost-effectiveness study in Indonesia found that scale-up of HCV treatment in the general population would be cost-effective with price reductions for HCV treatment and diagnostics, particularly when focusing treatment among PWID (Trickey et al., 2020). No studies from LMICs have modelled the HCV pathway-of-care among PWID for different service settings. These analyses are useful for giving guidance on the most cost-effective combination of HCV-related interventions for achieving elimination.

### Strengths and limitations

This study’s strengths include the use of data from the various detailed surveys and DRIVE-C (Des Jarlais et al., 2021; Nagot et al., 2023), as well as other data from PWID in Haiphong, Vietnam, such as population size estimations (Des Jarlais et al., 2018) and OAT uptake numbers. These detailed data were incorporated into a dynamic, deterministic mathematical model using a Bayesian framework, which allowed us to incorporate and account for data uncertainty in our model projections. We also utilised cost data collected or estimated from existing interventions, to enable a realistic estimation of the cost-effectiveness of different interventions. Additionally, the model generally agreed well with validation data, increasing confidence in our model projections.

Limitations included uncertainty in whether the number of PWID and ex-injectors in Haiphong is stable or decreasing. However, we believe the modelled decrease in the number of PWID over time is accurate due to the observed reduction in new injectors recruited in successive RDS survey rounds, with 27 % of respondents in 2016 survey having started injecting in the previous 4 years, compared with 9 % in 2019 survey. There was also uncertainty in other model parameters, which was incorporated into the model calibration and so the model projections. Based on data from DRIVE, the reinfection rate was assumed to be lower than the primary incidence rate. This could be partially due to a high proportion of this cohort being on OAT and so might not be representative of the entire population of PWID in this setting. Despite this, the primary and reinfection rates were calculated from the same sample, so the relative rate ratio used could still be robust. We assumed the same cost, treatment length, and monitoring for treatment of primary infections and reinfections, due to a lack of information on this. We could not incorporate all costs and benefits associated with improved HCV-related outcomes, such as reductions in HCV healthcare costs, as these were not available. Not including these costs gives us a conservative (less cost-effective) estimate of the impact of the intervention. In a sensitivity analysis where we included healthcare costs from Cambodia (Walker et al., 2020), the interventions became more cost-effective. However, the applicability of these costs to Vietnam is uncertain, as Cambodia’s GDP per capita in 2023 was around 57 % of Vietnam’s. Importantly, all modelled HCV testing and treatment interventions are cost-effective without this added cost saving being included. We did not explicitly model HIV transmission as this would have complicated the model. This simplification should not have affected our results because the incidence of HIV is now low in Haiphong (Moles et al., 2020). We did not model the impact of the COVID-19 pandemic on these HCV interventions in Haiphong, due to a lack of data. However, this is likely to have had a large effect on many aspects of life in Vietnam between 2020 and 2022 (Hoang, 2022).

### Implications

Our analysis shows that scaling up HCV testing and treatment interventions through RDS surveys and other service settings is a cost-effective strategy for getting close to achieving HCV elimination among PWID in Haiphong. With suitable funding and resources, this intervention scenario is likely to be feasible as these interventions have already been undertaken among PWID in Haiphong utilising links with community groups. This has been made achievable due to the availability of rapid point-of-care HCV antibody and RNA tests (Trickey et al., 2023; Vetter et al., 2022) and highly effective and affordable DAA treatment (Falade-Nwulia et al., 2017), with considerable contribution from peer support networks to aid the treatment scale-up. As provision of HCV testing and treatment at ART centres is limited to those people with HIV, the prevalence of which is decreasing over time in Haiphong, it is likely that the provision of testing and treatment in OAT centres has greater potential for achieving and maintaining future impact, especially if OAT coverage is increased. Due to this, future HCV testing strategies in Haiphong and elsewhere in Vietnam should transition to having more focus on OAT centres, which should be aided by ongoing reductions in HCV testing and treatment costs to make these initiatives more cost-effective. These interventions require ongoing support and investment from policy makers, both from national and international stakeholders. The collaboration between the Vietnam Administration for HIV/AIDS Control and the Global Fund is an example of this, but one that may be threatened by changes to aid budgets, particularly in the US (Brink et al., 2025). Policy makers should also consider the attrition from these interventions when individuals are imprisoned. This was a major source of loss-to-follow-up in the DRIVE-C study (Nagot et al., 2023). Future interventions should consider introducing HCV testing and treatment in prisons, as has been done successfully in other countries (Johnson et al., 2023). The rate of incarceration was high in this population, although the median duration of incarceration was low. A lack of continuation of these interventions in prison is likely to be a substantial source of attrition, reducing their effectiveness. For testing and treatment of HCV in OAT centres to be successful in other settings, the coverage of OAT will need to be increased to ensure this strategy reaches sufficient PWID, and similar strategies should be considered in other harm reduction interventions.

## Supplementary Material

Supplementary material Supplementary material associated with this article can be found, in the online version, at doi:10.1016/j.drugpo.2025.104898.

## Figures and Tables

**Table 1:** Model calibration and validation parameters† and other key model parameters with range in brackets.

Parameter	Value	Source
**Main model: calibration**	**Point estimate**	
Active PWID population size (2014)	5220	Des Jarlais et al., 2018(7)
Active PWID population size (2016)	4617	Des Jarlais et al., 2018(7)
Number of individuals on OAT (2011)	2065	OAT clinic data, Haiphong
Number of individuals on OAT (2014)	3407	OAT clinic data, Haiphong
Number of individuals on OAT (2017)	3924	OAT clinic data, Haiphong
Number of individuals on OAT (2019)	3898	OAT clinic data, Haiphong
Population size with history of injecting drug use (includes current) (2016)	10,000	Des Jarlais et al., 2018(7)
% of active PWID aged 40+ (2017)	43 %	RDS1
% of active PWID aged 40+ (2018)	44 %	RDS2
% of active PWID aged 40+ (2019)	52 %	RDS3
% of active PWID aged 40+ (2020)	55 %	RDS4
HIV prevalence among active PWID (2014)	25 %	DRIVE-IN(14)
HCV Antibody prevalence among active PWID (2017)	70 %	RDS1
HCV Antibody prevalence among active PWID (2018)	69 %	RDS2
HCV Antibody prevalence among active PWID (2019)	72 %	RDS3
HCV Antibody prevalence among active PWID (2020)	73 %	RDS4
HCV RNA prevalence among active PWID (2019)	60 %	RDS3
% of active PWID self-reporting ever being tested for HCV pre-RDS1 (2017)	22 %	RDS1
% of active PWID self-reporting ever being on OAT (2019)	56 %	RDS3
% of active PWID self-reporting ever being on OAT (2020)	60 %	RDS4
HCV Antibody prevalence among active PWID in the community who have never been incarcerated (2019)	66 %	RDS3
HCV Antibody prevalence among active PWID in the community who have ever been incarcerated (2019)	78 %	RDS3
Cumulative number of historical treatments (2020)	686	DRIVE-C
Cumulative number of historical treatments in OAT centres (2023)	191	VAAC-GF
Cumulative number of historical treatments in ART centres (2023)	640	VAAC-GF
Number of PWID in OAT centres with diagnosed HCV (2023)	333 more than 2021 value	VAAC-GF
Number of PWID in ART centres with diagnosed HCV (2023)	1000 more than 2021 value	VAAC-GF
**Incarceration mini-model: calibration**	**Point estimate**	
% of active PWID self-reporting as ever incarcerated 2.5 years after starting to inject	45 %	RDS3, RDS4
% of active PWID self-reporting as ever incarcerated 7.5 years after starting to inject	59 %	RDS3, RDS4
% of active PWID self-reporting as ever incarcerated 12.5 years after starting to inject	64 %	RDS3, RDS4
% of active PWID self-reporting as ever incarcerated 17.5 years after starting to inject	65 %	RDS3, RDS4
**Main model: validation**	**Point estimate (95 % CI)**	
% of active PWID self-reporting ever being tested for HCV pre-RDS2 (2018)	41 % (95 %CI: 39–44 %)	RDS2
% of active PWID self-reporting ever being tested for HCV pre-RDS3 (2019)	48 % (95 %CI: 46–51 %)	RDS3
% of active PWID self-reporting ever being tested for HCV pre-RDS4 (2020)	60 % (95 %CI: 57–62 %)	RDS4
% of active PWID self-reporting currently being on OAT (2017)	12 % (10–14 %)	RDS1
% of active PWID self-reporting currently being on OAT (2018)	32 % (30–35 %)	RDS2
% of active PWID self-reporting currently being on OAT (2019)	41 % (39–44 %)	RDS3
% of active PWID self-reporting currently being on OAT (2020)	49 % (46–52 %)	RDS4
% of active PWID self-reporting ever being incarcerated (2019)	65 % (95 %CI: 62 %–67 %)	RDS3
% of active PWID self-reporting ever being incarcerated (2020)	66 % (95 %CI: 63–68 %)	RDS4
Overall HCV incidence per 100 person-years (community) (2017)	8.9 (95 %CI: 7.2–11.1)	DRIVE cohort
Reinfection HCV incidence per 100 person-years (community) (2019)	4.1 (95 %CI: 2.0–7.3)	Nagot et al., 2023 (8)
% of active PWID self-reporting being released from prison in last 12 months (2019)	8.4 % (95 %CI: 7.4–9.5 %)	RDS3, RDS4
**Other key model prior parameters**	**Point estimate (Sampling Range)**	
Rate ratio for HCV incidence if HIV+	5.74 (3.44–9.59) (Triangular)	DRIVE data
Rate ratio for HCV incidence if aged 40+	0.53 (0.34–0.83) (Triangular)	DRIVE data
Rate ratio for HCV incidence if on OAT	0.50 (0.40–0.63) (Triangular)	(Platt et al., 2017)
Rate ratio for HCV incidence if recently released from prison	1.62 (1.28–2.05) (Triangular)	(Stone et al., 2018)
Rate ratio for HCV incidence if currently imprisoned	1 (0.5–2) (Triangular)	Estimated through model calibration
Incarceration rate per year	0.044 (0.023–0.067) Triangular	DRIVE data
Reincarceration rate per year	0.086 (0.058–0.122) Triangular	DRIVE data
Rate of release from prison per year (reflecting median distribution of incarceration)	4.02 (3.02–121.7) Triangular	DRIVE data

PWID: People who inject drugs. OAT: Opiate agonist therapy. RDS1–4: Respondent driven sampling surveys 1–4 in Haiphong, Vietnam. HCV: Hepatitis C virus. RNA: Ribonucleic acid. 95 %CI: 95 % confidence interval.

† All apply to PWID in the community unless specified.

**Table 2:** Historical interventions for HCV among PWID in Haiphong, and scenarios modelled.

Historical HCV intervention	Details
DRIVE-IN RDS survey	603 active injectors HCV Ab tested (2014)
RDS1 survey	1380 active injectors HCV Ab tested (2016)
RDS2 survey	1451 active injectors HCV Ab tested (2017)
RDS3 survey	1443 active injectors HCV Ab tested, of which 1039 were HCV positive and 1038 were RNA tested (as part of DRIVE-C)
DRIVE-C	1425 RNA tested from RDS3 and the DRIVE cohort created from the RDS surveys. Of these, 1201 were HCV RNA-positive, 1021 were eligible for HCV treatment and 979 initiated the HCV treatment
RDS4 survey	1267 active injectors HCV Ab tested (2019)
VAAC-GF intervention	In OAT centres, 333 diagnosed with HCV and 191 initiated HCV treatment; in ART centres, 1000 diagnosed and 640 initiated treatment (2021-2022). Both settings include active and former injectors.
**Scenario**	**Description**
Scenario 0a	No existing or future HCV testing and treatment interventions through RDS surveys and in OAT and ART centres (full counterfactual)
Scenario 0b	No DRIVE-C intervention (partial counterfactual) or future HCV testing and treatment interventions, but still include VAAC-GF intervention and historical RDS surveys.
Scenario 1	Status quo (historical interventions) until 2022 but no testing and treatment interventions over 2025–2030
Scenario 2	Scenario 1 plus HCV testing and treatment at ART and OAT centres over 2025–2030 among active and former injectors
Scenario 3	Scenario 1 plus annual RDS surveys among active injectors 2025–2030
Scenario 4	Scenario 1 plus annual RDS surveys among active injectors and HCV testing and treatment at ART and OAT centres from 2025–2030

PWID: People who inject drugs. HCV: Hepatitis C virus. OST: Opiate agonist therapy. ART: Antiretroviral therapy. RDS: Respondent-driven sampling. Ab: Antibody. RNA: Ribonucleic acid.

**Table 3:** Modelled overall community HCV incidence among PWID and ex-injectors in 2030, per 100 person-years, for each future scenario.

Scenario	HCV incidence in 2030, per 100 person-years (95 % credibility interval)	Percentage change in HCV incidence over 2015 to 2030 (95 % credibility interval)	Numbers of HCV treatment regimens administered over 2025–2030	Percentage of runs that achieve WHO’s HCV elimination target of <2 infections per 100 person-years among PWID
1: Status quo (SQ) until 2022 and nothing after that	6.2 (3.5–10.7)	25 % (9–37 %)	0 (0–0)	0.1 %
2: SQ plus HCV testing and treatment at ART and OAT centres from 2025 to 2030	4.8 (2.3–8.9)	43 % (26–59 %)	1608 (884–2596)	0.7 %
3: SQ plus annual RDS surveys 2025–2030	3.7 (1.5–8.1)	54 % (31–74 %)	1858 (814–3209)	8.2 %
4: SQ plus annual RDS surveys and HCV testing and treatment at ART and OAT centres from 2025 to 2030	2.7 (1.0–6.4)	67 % (46–84 %)	2986 (2035–4362)	29.0 %

HCV: Hepatitis C virus. OST: Opiate agonist therapy. ART: Antiretroviral therapy. RDS: Respondent-driven sampling.

**Table 4:** Cost-effectiveness projections of future intervention scenarios versus status quo scenario 1 and fully incremental cost-effectiveness analysis over 2025–2054.

Scenario	Incremental costs compared to scenario 1 (Euros)	Incremental costs compared to previous scenario (Euros)	DALYs averted compared to scenario 1	DALYs averted compared to previous	ICER compared to Status Quo scenario 1	ICER compared to previous
1: Status quo (SQ) until 2022 and nothing after that	0	NA	0	NA	NA	NA
2: SQ plus HCV testing and treatment at ART and OAT centres from 2025 to 2030	897,643	897,643	2188	2188	410	410
3: SQ plus annual RDS surveys 2025–2030	1836,619	938,976	1850	–338	993	Dominated
4: SQ plus annual RDS surveys and HCV testing and treatment at ART and OAT centres from 2025 to 2030	2488,612	651,993	2815	964	884	2536 (vs scenario 2)
