# Supplementary material for: Impact and cost-effectiveness of interventions to eliminate hepatitis C virus among people who inject drugs in Haiphong, Vietnam
Source: Int J Drug Policy. Author manuscript; Available in PMC 2026 Apr 9. (PMC7618992; doi:10.1016/j.drugpo.2025.104898)
Supplement: Supplementary material — associated with this article can be found, in the online version, at doi:10.1016/j.drugpo.2025.104898. [file EMS212832-supplement-Supplementary_material.docx]

**Impact and cost-effectiveness of interventions to eliminate hepatitis C virus among people who inject drugs in Haiphong, Vietnam**

**SUPPLEMENTARY MATERIALS**

**Model overview**

We developed a dynamic, deterministic model of hepatitis C virus (HCV) transmission among current and former injecting drug users in Haiphong, Vietnam. The model consists of two parts, [1] an incarceration mini-model, and [2] the main HCV model that is used to describe the epidemic.

**Incarceration mini-model structure**

The incarceration mini-model has the structure shown below in supplementary figure 1. This is used to produce the proportions entering into the main model in each incarceration category. This model is fitted to the % ever incarcerated at 2.5, 7.5, 12.5, and 17.5 years, which is parameterised from survey data: 45% after 2.5 years of injecting, 59% after 7.5 years, 64% after 12.5 years, and 65% after 17.5 years. The model is run until equilibrium. This model calibration also estimates the incarceration and reincarceration rates were calculated (along with 95% confidence intervals) using data on multiple incarcerations over time from the cohort of injectors that were recruited via the respondent driven sampling (RDS) surveys.

The calculations for the proportions starting in each compartment, relating to the parameters and their corresponding values in supplementary table 1 are:

Never incarcerated: †

Currently incarcerated: ‡(1-†)

Previously incarcerated (≥12 months ago): ꚛ(1-†-‡(1-†))

Recently incarcerated (<12 months ago): 1-†-(‡(1-†))-ꚛ(1-†-‡(1-†))

Where † is the proportion of PWID starting as never incarcerated

‡ is the proportion of those ever incarcerated, who start as currently incarcerated.

ꚛ is the proportion of those previously, but not currently, incarcerated, who were most recently incarcerated over 12 months ago

**Supplementary figure 1:** Incarceration status schematic

**Main HCV model structure**

The main HCV model starts in 1980 with a view to getting a stable HCV prevalence by the start of the period of analysis: 2010. The model is stratified by 6 levels of compartments: low/high-risk injecting status (low-risk, high-risk without HIV, high-risk with HIV) [not shown in schematics], age (16-39, ≥40) [not shown in schematics], current injecting and opiate agonist treatment (OAT) status (current injector never on OAT, current injector on OAT, current injector previously on OAT, former injector never on OAT, former injector on OAT, former injector previously on OAT – former injectors include those who have temporarily and permanently ceased injecting) [supplementary figure 2], incarceration status (never incarcerated, currently incarcerated, released from prison in the last 12 months, previously incarcerated but not in last 12 months) [supplementary figure 1], HCV infection, diagnosis and treatment status [supplementary figure 3], and disease progression status (F0, F1, F2, F3, F4, decompensated cirrhosis, hepatocellular carcinoma) [supplementary figure 4].

The high-risk injecting categories stratified by HIV status are used to capture the effect of HIV infection status being a strong risk factor for HCV and mortality, and to allow us to project the impact of interventions in antiretroviral therapy (ART) centres. However, we do not model HIV transmission because it is very low among people who inject drugs (PWID) in Haiphong due to high ART coverage, as well as needle and syringe provision (NSP) and OAT(1).

*Entering the model*

Individuals enter the model each year representing people starting injecting drug use. The number of individuals entering the model decreases over time, with a proportional reduction in number of people entering the model at two time points. The times when these changes occur, and the relative changes are both estimated during the model calibration. People enter the model in the age 16-39 category as current injectors that are not on OAT and have never been infected with HCV. A proportion of entrants enter each of the four incarceration states and as either low-risk, high-risk with no HIV, or high-risk with HIV. The proportion entering as high-risk remains steady over time (calibrated to historical levels of HIV prevalence), but the proportion of these high-risk injectors with HIV (calibrated to more recent levels of HIV prevalence) reduces over time, to mimic the effect of ART scale-up. This proportion of the high-risk injectors entering with HIV changes linearly between the introduction of ART in 2005 to 0% in 2019 when ART coverage was very high and HIV transmission almost non-existent(2, 3).

*Moving through the model*

Injecting risk categories were included because, based on our unpublished analyses of data from the DRIVE studies, HIV status was associated with increased HCV acquisition risk. This is likely due to increased injecting risk behaviours rather than HIV status and we wanted to capture this heterogeneity in transmission risk. For simplicity, we do not model people transitioning between the low and high-risk injecting behaviour states over time because people do not transition from being HIV-positive, which is our proxy marker of high-risk status. In the model, HIV also increases mortality. The HIV mortality rate is stable until 2005 when harm reduction interventions started in Vietnam(4). By 2016, it has decreased linearly down to 25% of the pre-2005 HIV mortality rate. This coincides with high ART coverage levels at the time of the first RDS (75% ART coverage)(2). By 2019, the HIV mortality rate then decreases further linearly down to 7% of the pre-2005 HIV mortality rate to reflect ART coverage of 93% at the time of the fourth RDS(2, 3). This HIV mortality rate then remains steady going forwards. This reduction in HIV mortality, along with changes in injecting drug use initiation, produces an ageing cohort as seen in the DRIVE data(2).

People age from the 16-39 to the ≥40 age category over time. Age impacts on background annual mortality rates; 0.00195688 for those aged 16-39 and 0.302 for those aged 40+, calculated from the United Nations World Population Prospects 2019(5). Based on our HCV incidence analyses of DRIVE data, HCV transmission risk is lower in the higher age group. To capture the observed temporal increase in the median age when people initiate injecting drug use (24 years old in 2009, 27 in 2013, and 30 in 2019)(2), the rate of transitioning between the age groups increases linearly between 2009 and 2013 and between 2013 and 2019, and remains the same thereafter.

Injecting status and OAT status are split into 6 categories, as shown in supplementary figure 2. This allows for temporary/permanent cessation of injecting and the starting, stopping, and restarting of OAT. Only current injectors can acquire and transmit HCV. Rates of starting and restarting OAT differ by injecting status. OAT affects injecting cessation/relapse rates, which are parameterised based on our analyses of transition rates between injecting and formerly injecting (and vice-versa) using the DRIVE cohort data. Current injectors have a higher mortality rate due to overdose, with mortality being reduced if they are on OAT(6). OAT also reduces the risk of HCV acquisition and transmission among current injectors and increases the rate of HCV antibody testing rates based on our analyses of data from RDS1.

**Supplementary figure 2:** Injecting and OAT status schematic


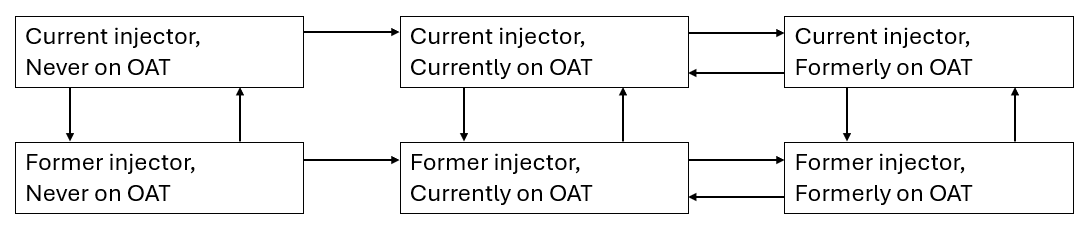


Regarding incarceration, the four compartments are the same as those shown in the mini-model – see supplementary figure 1. When incarcerated for the first time, people move from never incarcerated to currently incarcerated. Upon release, they move into the recently released (within the last year) category and then from there can be re-incarcerated or move to a previously incarcerated (but not recently) category, from where they can be re-incarcerated. Having been recently released from prison increases HCV transmission risk due to riskier behaviours upon release, as found in a recent systematic review(7) (supplementary table 1). We assume similar risk behaviours for PWID that have never been incarcerated, are currently incarcerated, or have been incarcerated >12 months ago. People who are incarcerated lose contact with OAT centres and RDS surveys, but can re-establish contact afterwards.

*HCV transmission, diagnosis, treatment, and progression*

There are 10 HCV infection, diagnosis, and treatment categories in the model as shown in supplementary figure 3. People start off as never infected and then, when infected, move to either the undiagnosed and uninfected (antibody (Ab)+ and ribonucleic acid (RNA)-) or undiagnosed and infected (Ab+ and RNA+) categories, depending on the proportion that spontaneous clear their infection. Depending on the rate of Ab testing, people can then move into the Ab known and RNA unknown categories. Similarly, depending on rates of RNA testing, people who have known Ab status but unknown RNA status can then move into categories for people with known Ab and RNA status. People Ab+, RNA- (known or unknown Ab+) can be re-infected and move into the undiagnosed infection (Ab+, RNA+) categories with either known or unknown Ab status. People in the diagnosed infection (Ab+ known, RNA+ known) category can initiate treatment. A small proportion of treatment is unsuccessful, so some people move from treatment back to the Ab+ known, RNA+ known category. People are assumed to be not infectious whilst on treatment because HCV is quickly suppressed under treatment(8). For successfully treated individuals, they move to a previously treated (Ab+ and RNA- known) category, from where they can be reinfected and move back into the Ab+ known, RNA+ unknown category.

The model assumes that HCV transmission only occurs between active PWID and does not occur between prison and the community. The HCV force of infection is dependent upon the HCV chronic prevalence among active PWID in each setting (i.e. prison or community) and is reduced for PWID on OAT or aged ≥40 but is increased among those who are high-risk and have recently been released from prison.

Testing and treatment mostly occur when historical and future interventions are added to the model. The only exception to this is that there is a low background level of Ab testing which is present before these interventions started (from 2010 onwards). This was seen in DRIVE-IN, with data suggesting it is increased among people on OAT. People in the Ab+ and RNA+ known category can be lost to follow-up and move to the Ab+ (known) and RNA+ (unknown) box at a rate calculated using data from RDS3 and DRIVE-C -ln((979/1349)/1.25). This is to account for people who have to be re-engaged through testing before they can engage in treatment if treatment is delayed after diagnosis. There is also a category for people who are diagnosed as HCV RNA+ whilst participating in an RDS, including those who knew their status beforehand. This is to allow us to separate out the effects of testing and treatment in the RDS from other future interventions (e.g. diagnosis in an OAT centre), as only people who have also been diagnosed through the RDS are treated through an RDS intervention. Once each RDS finishes, people return to the HCV RNA+ diagnosed (not in RDS) category.

**Supplementary figure 3:** Hepatitis C virus status schematic


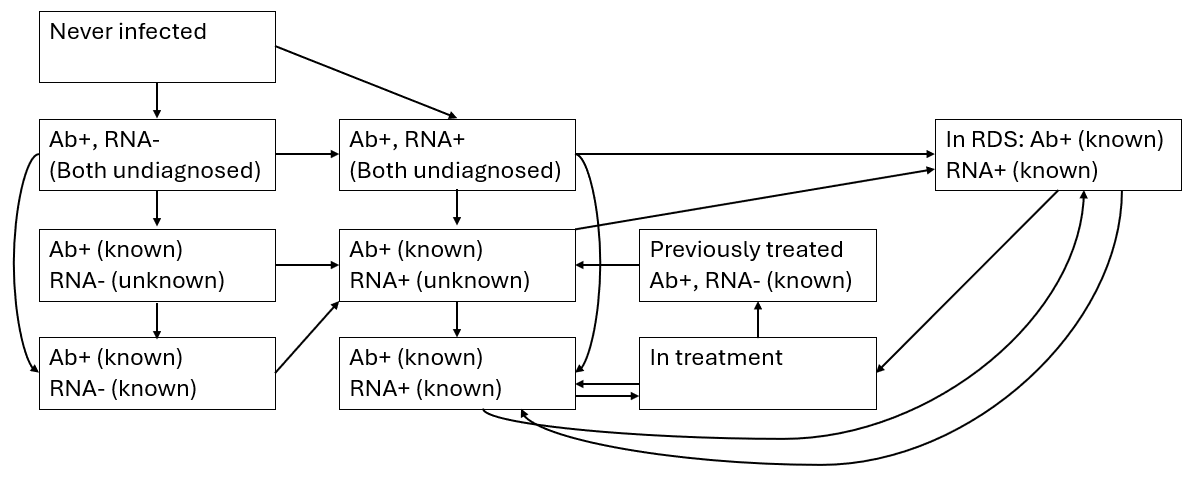
 Ab: Antibody. RNA: Ribonucleic acid. RDS: Respondent driven sampling survey.

Within each HCV infection status category there is HCV disease progression (fibrosis: F0, F1, F2, F3, F4, decompensated cirrhosis [DC], and hepatocellular carcinoma [HCC]), as shown in supplementary figure 4. There are separate, additional mortality rates for persons with DC and HCC. There is no progression in fibrosis for those HCV RNA-, except those with F4 or DC, where it progresses at a reduced rate. There is no disease progression whilst on treatment.

**Supplementary figure 4:** HCV disease progression schematic


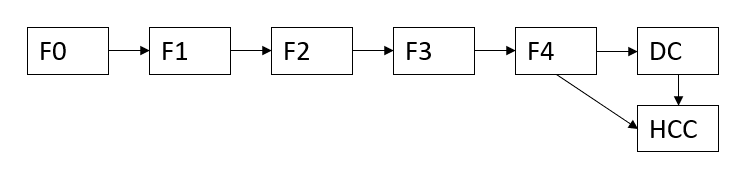


*Model parameterisation, calibration, and validation*

Most data used to parameterise and calibrate the model were taken from the 4 RDS surveys of current injectors that happened in Haiphong between 2016 and 2019 [N=1380, 1451, 1443, and 1267, respectively](2), as well as the DRIVE-IN survey from 2014 [N=603](4), and the DRIVE-C RDS from 2019 [N=1425](9). Data from the DRIVE cohort, recruited through the RDS surveys, were also used, whilst data on the numbers of people on OAT were taken from OAT clinic data in Haiphong. Additionally, some parameters were taken from the literature where Haiphong- or Vietnam-specific data were unavailable, particularly relating to HCV disease progression. Data from DRIVE-IN, the 4 RDS surveys, and DRIVE-C were also used to give the number of people captured and tested by each survey. We also included increased linkage to OAT when the surveys were occurring, as this was one of the goals of the DRIVE program, and the increase in the proportion of active injectors reporting being on OAT between RDS surveys reflects this(2). This is included in the model by having a rate of starting OAT, which is multiplied by a proportion (estimated during the model calibration) when the survey interventions are not occurring. All the surveys included HCV antibody testing, whilst RDS3 and DRIVE-C also included HCV RNA testing (2, 9). DRIVE-C additionally provided one of only two sources of HCV treatment in Haiphong to date, for 979 current and former injectors(9), the other being the VAAC-GF project in OAT and ART centres, which treated 191 people with HCV in OAT centres and 640 in ART centres (although not all will be current and former injectors). The number of current and former injectors diagnosed with HCV in OAT centres in the VAAC-GF intervention was around 1000 and was around 333 in ART centres. Rates of transition between incarceration states are calculated using data from the DRIVE cohort. The prior parameter ranges for the model are given in supplementary table 1.

The first step in the calibration process uses an incarceration sub-model that is calibrated to the percentage of active injectors that have ever been incarcerated in the four RDS surveys, by injecting duration (at 2.5, 7.5, 12.5, and 17.5 years of injecting). This is used to produce the proportions entering into the main model in each of the four incarceration categories. This sub-model follows a simulated cohort of 1000 PWID, who are followed for 17.5 years. The sub-model used Approximate Bayesian computation sequential Monte Carlo (ABC SMC) scheme to obtain a sample of 1000 incarceration-related parameter sets.

We assumed a multiplier for reinfection rates of 0.46 of the primary incidence rates, calculated as the 4.1 per 100 person-years reinfection rate from Nagot et al(9) divided by the overall incidence rate of 8.9 per 100 person-years from the DRIVE cohort.

We calibrated the model using an ABC SMC scheme to data from RDS surveys 1-4 and PWID population size estimates for Haiphong from Des Jarlais et al(10) (table 1). This included population size estimates (for community PWID not in prison) for the number of active injectors in 2014 and 2016, the number of individuals on OAT in 2011, 2014, 2017, and 2019, and the population size of individuals with a history of drug use in 2016. The data used for model calibration from the RDS surveys for PWID in the community were the percentage of active injectors aged ≥40 in each RDS, the HCV antibody prevalence among active injectors in each RDS, the HCV RNA prevalence among active injectors in 2019, the percentage of active injectors self-reporting being antibody tested before RDS1 (assumed to be equivalent to the percentage of individuals with HCV antibodies who have been diagnosed as being Ab+), and the percentage of active injectors self-reporting as currently or ever on OAT in RDS3 and RDS4. The model was calibrated to the HCV prevalence for PWID in the community, separately among those who have never and ever been incarcerated, which allows us to estimate the relative risk of transmission during periods of incarceration compared to when they are in the community. We also calibrated to the HIV prevalence among PWID in 2014, taken from DRIVE-IN and the HCV antibody prevalence in 2020 among active injectors who had never and ever been incarcerated, respectively, against their prevalence estimates from RDS4. We calibrated to the cumulative number of people treated by 2020, and the cumulative number of people treated by 2023 in OAT centres and ART centres, with these numbers taken from the DRIVE-C and VAAC-GF interventions. Also, from the VAAC-GF intervention, we calibrated to the number of current and former injectors in ART centres who had diagnosed HCV in 2023; setting this number as 1000 higher than the value in 2021. Similarly, for OAT centres we used a value of those diagnosed as 333 higher in 2023 than that in 2021. Setting these numbers as 1000 and 333 higher than the 2021 values was done to incorporate the testing and treatment numbers from the VAAC-GF intervention, whilst also generating estimates for the rates of diagnosis for these historical interventions that could be applied to future interventions.

The ABC SMC begins with 1000 parameter sets sampled from prior distributions (supplementary table 1), which are successively perturbed to improve their goodness of fit (measured as the sum of the relative errors to the calibration data). At each iteration, perturbed parameters sets are accepted if their goodness of fit falls below a certain tolerance, which is set adaptively to be the 75^th^ percentile of the goodness of fit scores from the previous iteration’s accepted parameter sets. We stopped the ABC SMC when the change in tolerances between iterations was less than 3% in 3 consecutive iterations – indicating little improvement in the goodness of fits between successive iterations. This produced 1000 baseline model fits, which were used for all model analyses to produce a median estimate and 95% credibility intervals around that estimate, defined as the 2.5^th^-97.5^th^ percentile range. The model of ordinary differential equations was solved using the Runge-Kutta formula[REF], with the solution outputted at every 0.1 year.

The baseline model fits for the percentage of active injectors with HCV antibodies that had been antibody diagnosed for HCV were cross-validated against the percentage of active injectors self-reporting as being tested for HCV in RDS2, RDS3, and RDS4 (table 1), which assumes the percentage tested will be equivalent to the percentage diagnosed. Validation was also performed by comparing the percentage of active injectors self-reporting as being on OAT in each survey with the percentage of active injectors in the model who are on OAT. We also validated the model projections against estimates from RDS3 and RDS4 on the proportion of active injectors self-reporting ever being incarcerated and recently released from prison. Additional validation was performed by comparing the model projections against estimates of the community primary and reinfection HCV incidence among PWID in the DRIVE cohort in 2017 and 2019(9), respectively.

**Model parameters**

Rates of incarceration and re-incarceration, and the median duration of incarceration were calculated using data from the DRIVE cohort. Mortality rates were also calculated through the DRIVE study as a combination of HCV- and HIV-related mortality, as well as other mortality among PWID (including overdoses). Additionally, some parameters were taken from the literature where Haiphong- or Vietnam-specific data were unavailable, particularly relating to HCV disease progression.

**Supplementary table 1:** Prior distributions and posterior distributions and sources for model parameters.

| **Parameter** | **Prior range** | **Source** | **Posterior median (range)** |
| --- | --- | --- | --- |
| **Starting assumptions** |  |  |  |
| Number of individuals starting in the model | 4000-20000 (Uniform) | NA | 6068 (4000-15138) |
| Proportion of individuals starting in the model as active injectors | 0-1 (Uniform) | NA | 0.57 (0.18-0.94) |
| Proportion of individuals starting in the model age 40+ | 0-1 (Uniform) | NA | 0.61 (0.01-0.84) |
| Proportion of individuals starting in the model as high risk | 0.48-0.68 (Uniform) | (4) Sampling between 2006 and 2009 HIV prevalence estimates due to uncertainty about reliability | 0.54 (0.48-0.63) |
| Starting HCV prevalence in the model (proportion) | 0-1 (Uniform) | NA | 0.38 (0.00-0.74) |
| **Recruitment** |  |  |  |
| The number of individuals entering the model each year (Early era) | 1-1000 (Uniform) | NA | 440 (269-607) |
| Mid era recruitment reduction proportion multiplier | 0-1 (Uniform) | NA | 0.74 (0.42-0.99) |
| Late era recruitment reduction proportion multiplier | 0-1 (Uniform) | NA | 0.75 (0.38-0.99) |
| Mid era start year | 2002-2015 (Uniform) | NA | 2005.6 (2002.2-2010.5) |
| Years late era starts after mid era | 1-16 (Uniform) | NA | 8.2 (3.0-15.9) |
| **Starting/stopping injecting** |  |  |  |
| Annual rate of ceasing injecting if never on OAT | 0.31 (0.27-0.36) Triangular | Calculated from DRIVE data | 0.30 (0.27-0.35) |
| Annual rate of ceasing injecting if on OAT | 0.57 (0.52-0.63) Triangular | Calculated from DRIVE data | 0.60 (0.56-0.63) |
| Annual rate of ceasing injecting if previously (but not currently) on OAT | 0.20 (0.11-0.39) Triangular | Calculated from DRIVE data | 0.27 (0.19-0.38) |
| Annual rate of restarting injecting if never on OAT | 0.46 (0.35-0.59) Triangular | Calculated from DRIVE data | 0.48 (0.39-0.59) |
| Annual rate of restarting injecting if on OAT | 0.52 (0.45-0.59) Triangular | Calculated from DRIVE data | 0.50 (0.46-0.55) |
| Annual rate of restarting injecting if previously (but not currently) on OAT | 0.34 (0.18-0.66) Triangular | Calculated from DRIVE data | 0.36 (0.21-0.57) |
| **Starting/stopping OAT** |  |  |  |
| Annual rate of starting OAT if currently injecting 2016- | 0.34 (0.29-0.40) Triangular | Calculated from DRIVE data | 0.32 (0.30-0.38) |
| Annual rate of starting OAT if not currently injecting 2016- | 0.11 (0.07-0.17) Triangular | Calculated from DRIVE data | 0.11 (0.07-0.15) |
| Annual rate of stopping OAT if currently injecting 2016- | 0.15 (0.12-0.18) Triangular | Calculated from DRIVE data | 0.15 (0.13-0.17) |
| Annual rate of stopping OAT if not currently injecting 2016- | 0.07 (0.05-0.10) Triangular | Calculated from DRIVE data | 0.07 (0.05-0.09) |
| Annual rate of restarting OAT if currently injecting 2016- | 0.68 (0.47-0.99) Triangular | Calculated from DRIVE data | 0.69 (0.47-0.86) |
| Annual rate of restarting OAT if not currently injecting 2016- | 0.34 (0.18-0.66) Triangular | Calculated from DRIVE data | 0.26 (0.18-0.51) |
| Rate ratio of starting OAT 2008-15 (vs 2016-) | 0-1 (Uniform) | NA | 0.31 (0.02-0.74) |
| Rate ratio of restarting OAT 2008-15 (vs 2016-) | 0-1 (Uniform) | NA | 0.18 (0.00-0.51) |
| **Incarceration mini model** |  |  |  |
| † Proportion of PWID starting as never incarcerated | 0.53-1 (Uniform) | Calculated from DRIVE data | 0.66 (0.53-0.92) |
| ‡ Proportion of those ever incarcerated, who start as currently incarcerated. | 0-1 (Uniform) | NA | 0.48 (0.11-0.87) |
| ꚛ Proportion of those previously, but not currently, incarcerated, who were most recently incarcerated over 12 months ago | 0-1 (Uniform) | NA | 0.76 (0.44-1.00) |
| **Incarceration** |  |  |  |
| Incarceration rate per year | 0.044 (0.023-0.067) Triangular | Calculated from DRIVE data | 0.05 (0.04-0.07) |
| Reincarceration rate | 0.086 (0.058-0.122) Triangular | Calculated from DRIVE data | 0.09 (0.07-0.12) |
| Rate of release from prison | 4.02 (3.02-121.7) Triangular | Calculated from DRIVE data: median incarceration duration: 90 days (IQR: 3-120) | 21.85 (3.15-56.73) |
| **HCV testing/treatment/incidence** |  |  |  |
| Anti-HCV background testing rate 2010 onwards | 0-0.25 (Uniform) | NA | 0.19 (0.00-0.25) |
| Relative increase in HCV testing if on OAT | 2.33 (1.65-3.29) (Triangular) | Calculated from DRIVE data | 2.53 (1.83-3.05) |
| DAA treatment efficacy | 91.7% (90-94%) (Triangular) | (9) | 0.93 (0.92-0.94) |
| % infections that spontaneous clear | 17% (10-40%) Triangular | Calculated from RDS3 data | 0.34 (0.22-0.40) |
| Rate ratio for HCV incidence if “high-risk” | 5.74 (3.44-9.59) (Triangular) | Calculated from DRIVE data | 7.73 (4.99-9.58) |
| Rate ratio for HCV incidence if aged 40+ | 0.53 (0.34-0.83) (Triangular) | Calculated from DRIVE data | 0.45 (0.34-0.71) |
| Rate ratio for HCV incidence if on OAT | 0.50 (0.40-0.63) (Triangular) | (11) | 0.56 (0.40-0.63) |
| Rate ratio for HCV incidence if recently imprisoned | 1.62 (1.28-2.05) (Triangular) | (7) | 1.68 (1.42-2.04) |
| Rate ratio for HCV incidence if currently imprisoned | 1 (0.5-2) (Triangular) | NA | 1.54 (0.77-2.00) |
| HCV Beta | 0-0.5 (Uniform) | NA | 0.12 (0.04-0.22) |
| HCV treatment rate during the DRIVE-C intervention | 0-1.035 (Uniform) | Calculated from DRIVE-C data, (-ln(1-(979/1349))/1.25) | 0.40 (0.13-0.82) |
| HCV testing rate in OAT centres during the VAAC-GF intervention | 0-4 (Uniform) | NA | 0.55 (0.01-2.23) |
| HCV testing rate in ART centres during the VAAC-GF intervention | 0-4 (Uniform) | NA | 3.24 (0.62-4.00) |
| HCV treatment rate in ART centres in the VAAC-GF intervention | 0-4 (Uniform) | NA | 1.21 (0.21-3.02) |
| HCV treatment rate in OAT centres in the VAAC-GF intervention | 0-4 (Uniform) | NA | 0.29 (0.00-1.98) |
| Rate of LTFU from treatment | 0-0.256 (Uniform) | Calculated from DRIVE-C data, -ln((979/1349))/1.25 | 0.20 (0.07-0.26) |
| **HCV disease progression parameters** |  |  |  |
| Annual rate of progression from F0 to F1 in HIV negatives with chronic HCV infection | 0.128 (95%CI: 0.08-0.176) Triangular | (12) | 0.10 (0.08-0.17) |
| Annual rate of progression from F1 to F2 in HIV negatives with chronic HCV infection | 0.059 (95%CI: 0.035-0.082) Triangular | (12) | 0.05 (0.04-0.08) |
| Annual rate of progression from F2 to F3 in HIV negatives with chronic HCV infection | 0.079 (95%CI: 0.056-0.10) Triangular | (12) | 0.09 (0.06-0.10) |
| Annual rate of progression from F3 to F4 in HIV negatives with chronic HCV infection | 0.116 (95%CI: 0.07-0.161) Triangular | (12) | 0.12 (0.10-0.15) |
| Annual probability of progression to decompensated cirrhosis from F4 in individuals with chronic HCV infection | 0.039 | (13) Beta(14.6168,360.1732) | 0.01 (0.00-0.04) |
| Annual probability of progression to HCC from F4 or decompensated cirrhosis in individuals with chronic HCV infection. | 0.014 | (13) Beta(1.9326,136.1732) | 0.04 (0.01-0.07) |
| Relative risk for progression from compensated to decompensated cirrhosis following SVR | 0.07 (95%CI 0.03-0.2) Triangular | (14) | 0.06 (0.03-0.12) |
| Annual probability of death from decompensated cirrhosis | 0.13 | (13) Beta(147.03,983.97) | 0.11 (0.07-0.14) |
| Relative risk for progression rate from compensated cirrhosis to HCC following SVR | 0.23 (95%CI 0.16-0.35) Triangular | (15) | 0.20 (0.16-0.26) |
| Annual probability of death from HCC | 0.43 | (13) Beta(117.1033,155.23) | 0.26 (0.20-0.34) |
| **Non-HCV-related mortality** |  |  |  |
| Non-HIV or HCV-related mortality rate per year among PWID | 0.015 (0.012-0.018) (Triangular) | (16) All mortality among HIV-ve PWID except that due to liver-related causes | 0.02 (0.01-0.02) |
| Relative risk of all-cause mortality if on OAT (current injectors) | 0.33 (95%CI: 0.28-0.39) Triangular | (17) Lognormal distribution | 0.31 (0.28-0.39) |
| Pre-2005 HIV mortality rate per year | 0.255 (0.131-0.328) (Triangular) | (18) | 0.02 (0.01-0.03) |

*Cost-effectiveness analysis*

The incremental cost-effectiveness ratio (ICER) of the status quo scenario 1 was firstly estimated in terms of costs (in 2023 Euros) per disability adjusted life years (DALYs) averted over 30 years (2014-2043) compared with the counterfactual scenarios 0a and 0b. The ICER of all future HCV testing and treatment scenarios were then estimated over 30 years (2025-2054) compared with the status quo scenario (scenario 1). A discount rate of 3% per year was applied to all DALYs and costs with a base year of 2023. Costs and DALY weights were applied to model compartments as described below. For each of the 1000 model fits, an ICER was calculated as the incremental costs divided by incremental DALYs, with mean ICERs and DALYs across model runs reported. A fully incremental cost-effectiveness analysis was also conducted which involves ordering the intervention scenarios in terms of increasing cost, and calculating incremental cost and DALYs averted, eliminating any interventions which are dominated (higher cost and fewer benefits) or extendedly dominated (higher ICER and fewer benefits). These comparisons were done to determine the most cost-effective intervention scenario. Cost-effectiveness was evaluated using a willingness-to-pay (WTP) threshold of 57% of Vietnam’s per capita GDP in 2023 (4,095 Euros, 57% is €2334)(19, 20), and cost-effectiveness acceptability curves (CEAC) presented to show how choice of scenario varies by WTP threshold.

DALYs were calculated using disability weights assigned to liver disease states in the model. Disability weights were taken from the 2019 Global Burden of Disease study(21), with some assumptions made where weights were not available for a disability state included in our model – see supplementary table 2. For assessing the cost-effectiveness of the status quo scenario 1, we included costs for RDS3 and DRIVE-C interventions (including HCV antibody and RNA testing), HCV RNA and antibody testing in OAT and ART centres, and HCV treatment costs (supplementary table 3). For assessing the cost-effectiveness of each future scenario, we similarly included the costs of the included future interventions. Costs were estimated from the perspective of an external investor (e.g. a grant) adding HCV testing and treatment to existing services and/or undertaking RDS surveys, rather than from the government, so healthcare costs avoided were not included. However, because the RDS surveys also improve referral to OAT, and it is possible that governments may undertake these interventions, we also undertook sensitivity analyses where the cost-effectiveness projections also included the costs of OAT. We also performed a sensitivity analysis using disability weights from the 2021 Global Burden of Disease study(22); which included disability weights corresponding to active injecting drug use and being on OAT; the 2021 weight for compensated cirrhosis was 0, whilst it was 0.114 in 2019 (supplementary table 4). In a separate sensitivity analysis we included healthcare costs estimated from a study of HCV treatment in Cambodia(23) (given in supplementary table 5).

We conducted expected value of partial perfect information (EVPPI) analysis to identify the model parameters with the greatest influence on the cost-effectiveness outcome in the base case. This was conducted using the BCEA package in R using the function *info.rank* which plots the ratio of EVPPI against the total expected value of perfect information (EVPI) for the willingness to pay threshold(24). This represents the relative importance of each parameter in terms of its impact on the decision of which intervention is most cost-effective.

*Cost estimation*

The DRIVE-C intervention enrolled patients diagnosed with HCV through the RDS or an ongoing cohort study on treatment. At initiation, patients were given direct acting antivirals (DAAs: sofosbuvir 400-mg/day and daclatasvir 60-mg/day). Cirrhotic patients were initially treated with 12 weeks of sofosbuvir/daclastavir plus ribavirin, and in case of problems were transitioned to sofosbuvir/daclatasvir for 24 weeks. At treatment and follow up visits, patients were clinically evaluated, had blood collected for laboratory tests and biobanking, received counselling on HCV treatment and adherence(9). Costs were estimated from initial screening date to treatment success evaluation after HCV treatment completion (24 weeks). Resources incurred included: laboratory testing, imaging (ultrasound/fibroscan), HCV medications, hospital staff, hospital space and overhead, equipment and supplies, education, and outreach. Anonymised patient-level data on number and types of study visits, laboratory tests, and drugs were taken from study records to calculate patient-level resource use. We assumed the same cost, treatment length, and monitoring for treatment of primary infections and reinfections, due to a lack of information on this.

We estimate the unit cost of HCV screening and treatment accounting for overheads, staff time, equipment, and consumables. Expenditure data from DRIVE-C and RDS screening were collected from 2018 and 2019 (18 months) excluding research related costs such as bio-banking and clinical trial-specific costs. Cost data for HCV screening and treatment were gathered retrospectively during an in-person costing exercise in February 2020 through interviewing study management and expenditure record review. The cost per person for the RDS surveys and GeneXpert were calculated separately from treatment costs. All costs are presented in 2023 Euros. Costs were collected in 2018 and 2019 based on expenditure data within the project, which was either in Vietnamese Dong or Euros. Costs were converted by first inflating to 2019 values based on Vietnam consumer price index (CPI: <https://data.worldbank.org/indicator/FP.CPI.TOTL?locations=VN>) and then converting Dong  to Euros using the 2019 exchange rate of 26,311 Dong per Euro. Costs in 2019 Euros were then further inflated to 2023 Euro values based on Vietnam CPI.

Expenditure data were collated from staff salary sheets, hospital budgets and study invoices and allocated to the relevant cost categories using the Healthcare Cost and Outcomes Model (HCOM) (<http://www.heroza.org/researchtools/testing-platform-cost-model>), accounting for patient-level resource use. The cost calculation allocates indirect costs such as staff costs, buildings, annualised equipment costs, supplies, and other fixed costs, while direct costs consist of staff time/event costs, lab costs, drugs, and patient transport.

Costs of OAT and ART were used from previously published studies. The cost of DAAs was updated to reflect reductions in treatment costs and is modelled with future treatment costs lower than historical treatment costs(25).

**Supplementary table 2:** Disability weights applied to each disease progression category, taken from the 2019 Global Burden of Disease study(21)

| **Disease progression stage** | **Disability weight value** |
| --- | --- |
| F0 | 0 |
| F1 | 0.25 * F4 value |
| F2 | 0.5 * F4 value |
| F3 | 0.75 * F4 value |
| F4 | Triangular distribution: (0.078, 0.114, 0.159) |
| DC | Triangular distribution: (0.1213, 0.178, 0.25) |
| HCC | Triangular distribution: (0.307, 0.451, 0.6) |

**Supplementary table 3:** Unit costs per item

| **Item** | **Cost (Euros)** |
| --- | --- |
| RDS survey per person | 119 |
| HCV Antibody test | 3 |
| HCV GeneXpert (RNA) test | 23.32 |
| Opiate substitution therapy cost | 401.51 |
| Historical costs for HCV treatment | 658 |
| Future costs for HCV treatment* | 470 |

*Using a Global Fund sofosbuvir/daclastavir cost of 100 euros.

**Supplementary table 4:** Disability weights applied to each disease progression category, taken from the 2021 Global Burden of Disease study(22), used in sensitivity analyses.

| **Disability Weights** | **Disability weight value** |
| --- | --- |
| Compensated cirrhosis (F4)* | 0 |
| Decompensated cirrhosis | Triangular distribution: (0.1213, 0.178, 0.25) |
| Hepatocellular carcinoma | Triangular distribution: (0.307, 0.451, 0.6) |
| Baseline for active PWID | Triangular distribution: (0.510, 0.697, 0.843) |
| Active PWID on methadone | Triangular distribution: (0.221, 0.335, 0.473) |

*Assuming 0 for less advanced fibrosis states (F0, F1, F2, F3).

**Supplementary table 5:** Healthcare costs taken from a study of HCV treatment in Cambodia(23), used in sensitivity analyses.

| **Disease stage** | **Associated healthcare cost**  **(in 2017 USD)** | **Associated healthcare cost**  **(in 2023 Euros)** |
| --- | --- | --- |
| F0 | 0 | 0 |
| F1 | 39.06 | 44.09 |
| F2 | 88.25 | 107.91 |
| F3 | 149.74 | 183.1 |
| F4 | 226.42 | 276.86 |

*Inflated using consumer price index from the USA between 2017 and 2023 (1.28) and then converted to Euros using 2023 exchange rate of (0.953 Euros per USD)

**Supplementary figure 5:** Number of all PWID and former injectors in Haiphong, the number of active injectors (aPWID), and the percentage of active injectors aged ≥40 in the status quo scenario.

Black lines are the median estimates and the purple area represents the 95% credibility intervals.

Grey dots: parameters used for calibration (vertical black lines are intervals)

**Supplementary figure 6:** Number of all PWID and former injectors in Haiphong on opiate agonist treatment (OAT), the percentage of active injectors (aPWID) who have ever been on OAT, and the percentage of active injectors on OAT in the status quo scenario.

Grey dots: parameters used for calibration (vertical black lines are intervals)

Green dots: parameters used for validation (vertical black lines are intervals)

Black lines are the median estimates and the purple area represents the 95% credibility intervals.

**Supplementary figure 7:** The percentage of active injectors (aPWID) in Haiphong with HCV antibodies (Ab+), the percentage of active injectors with HCV antibodies who have been diagnosed, the overall community HCV incidence rate per 100 person-years, and the reinfection rate in the status quo scenario

Grey dots: parameters used for calibration (vertical black lines are intervals)

Green dots: parameters used for validation (vertical black lines are intervals)

Black lines are the median estimates and the purple area represents the 95% credibility intervals.

**Supplementary figure 8:** The percentage of active injectors (aPWID) who have ever been incarcerated (ever pris), the percentage of current and former injectors who have recently been incarcerated (recently pris), the death rate per 100 person-years (100py) among current and former injectors, and the HIV prevalence among active injectors in the status quo scenario.

Grey dots: parameters used for calibration (vertical black lines are intervals)

Green dots: parameters used for validation (vertical black lines are intervals)

Black lines are the median estimates and the purple area represents the 95% credibility intervals.

**Supplementary figure 9:** Community HCV reinfection incidence per 100 person years

**Supplementary figure 10:** HCV antibody (Ab) prevalence among active PWID in the community who have never been incarcerated (left) and those who have ever been incarcerated (right) in the status quo scenario.

Grey dots: parameters used for calibration (vertical black lines are intervals)

Black lines are the median estimates and the purple area represents the 95% credibility intervals.

**Supplementary figure 11:** HCV RNA prevalence among active PWID (aPWID) in the community (top left), the number of treatments ever given among PWID and ex-injectors overall (top right), in ART centres (bottom left), and in OAT centres (bottom right), all in the status quo scenario.

Grey dots: parameters used for calibration

Black lines are the median estimates and the purple area represents the 95% credibility intervals.

ART: Antiretroviral Therapy. OAT: Opiate agonist treatment.

**Supplementary table 5:** Incremental cost-effectiveness ratio (ICER) per disability adjusted life year (DALY) gained over 2014-2043 for the status quo (scenario #1) versus the counterfactual with no historical interventions (scenario #0a)

| **Scenario** | **Total costs (Euros)** | **Incremental total costs (Euros)** | **Total costs (Euros) of treatment** | **Total costs (Euros) of surveys** | **Total costs (Euros) of testing** | **DALYs**  **averted** | **ICER: Euros per DALY averted** |
| --- | --- | --- | --- | --- | --- | --- | --- |
| 0a: no historical interventions (counterfactual) | 0 | NA | 0 | 0 | 0 | 0 | 0 |
| 1: Baseline assumptions | 2,516,512 | 2,516,512 | 1,547,959 (62% of total) | 731,136 (29% of total) | 237,417 (9% of total) | 1979 | 1271 |

**Supplementary table 6:** Incremental cost-effectiveness ratio (ICER) per disability adjusted life year (DALY) gained over 2014-2043 for the status quo (scenario #1) versus the counterfactual with no RDS3 or DRIVE-C (scenario #0b)

| **Scenario** | **Total costs (Euros)** | **Incremental total costs (Euros)** | **Total costs (Euros) of treatment** | **Total costs (Euros) of surveys** | **Total costs (Euros) of testing** | **DALYs**  **averted** | **ICER: Euros per DALY averted** |
| --- | --- | --- | --- | --- | --- | --- | --- |
| 0b: no historical RDS3 or DRIVE-C survey interventions (counterfactual) | 1,574,624 | NA | 761,811 | 559,419 | 253,394 | N/A | N/A |
| 1: Baseline assumptions | 2,516,512 | 941,888 | 1,547,959 | 731,136 | 237,417 | 886 | 1063 |

**Supplementary table 7:** Incremental cost-effectiveness ratio (ICER) per disability adjusted life year (DALY) gained for each future intervention scenario for 2025-2054 compared with the status quo (scenario #1) from the perspective of an external investor.

| **Scenario** | **Total costs (Euros)** | **Incremental total costs (Euros)** | **Incremental costs (Euros) of treatment** | **Increments costs (Euros) of surveys** | **Increments costs (Euros) of testing** | **DALYs**  **averted** | **ICER: Euros per DALY averted** |
| --- | --- | --- | --- | --- | --- | --- | --- |
| 1: Status quo until 2022 and nothing after that | 0 | NA | NA | NA | NA | NA | NA |
| 2: HCV testing and treatment at ART and OAT centres from 2025-2030 | 897,643 | 897,643 | 715,686 | 0 | 181,957 | 2188 | 410 |
| 3: Annual RDS surveys 2025-2030 | 1,836,619 | 1,836,619 | 803,918 | 902,504 | 130,197 | 1850 | 993 |
| 4: Annual RDS surveys and HCV testing and treatment at ART and OAT centres from 2025-2030 | 2,488,612 | 2,488,612 | 1,304,412 | 902,504 | 281,696 | 2815 | 884 |

HCV: Hepatitis C virus. OAT: Opiate agonist therapy. ART: Antiretroviral therapy. RDS: Respondent driven sampling.

**Supplementary table 8: Current and former injecting drug users in Haiphong with decompensated cirrhosis and hepatocellular carcinoma in 2030 for each future intervention scenario, with 95% credibility intervals.**

| **Scenario** | **People with decompensated cirrhosis in 2030** | **People with hepatocellular carcinoma in 2030** | **Deaths (all causes) in 2030** |
| --- | --- | --- | --- |
| 1: Status quo until 2022 and nothing after that | 31 (6, 86) | 55 (22, 96) | 302 (230, 377) |
| 2: HCV testing and treatment at ART and OAT centres from 2025-2030 | 25 (5, 74) | 40 (15, 75) | 298 (227, 372) |
| 3: Annual RDS surveys 2025-2030 | 27 (5, 77) | 43 (16, 85) | 292 (223, 365) |
| 4: Annual RDS surveys and HCV testing and treatment at ART and OAT centres from 2025-2030 | 23 (4, 65) | 33 (11, 66) | 289 (221, 361) |

**HCV: Hepatitis C virus. OAT: Opiate agonist therapy. ART: Antiretroviral therapy. RDS: Respondent driven sampling.**

**Supplementary figure 12:** Cost-effectiveness acceptability curve

**
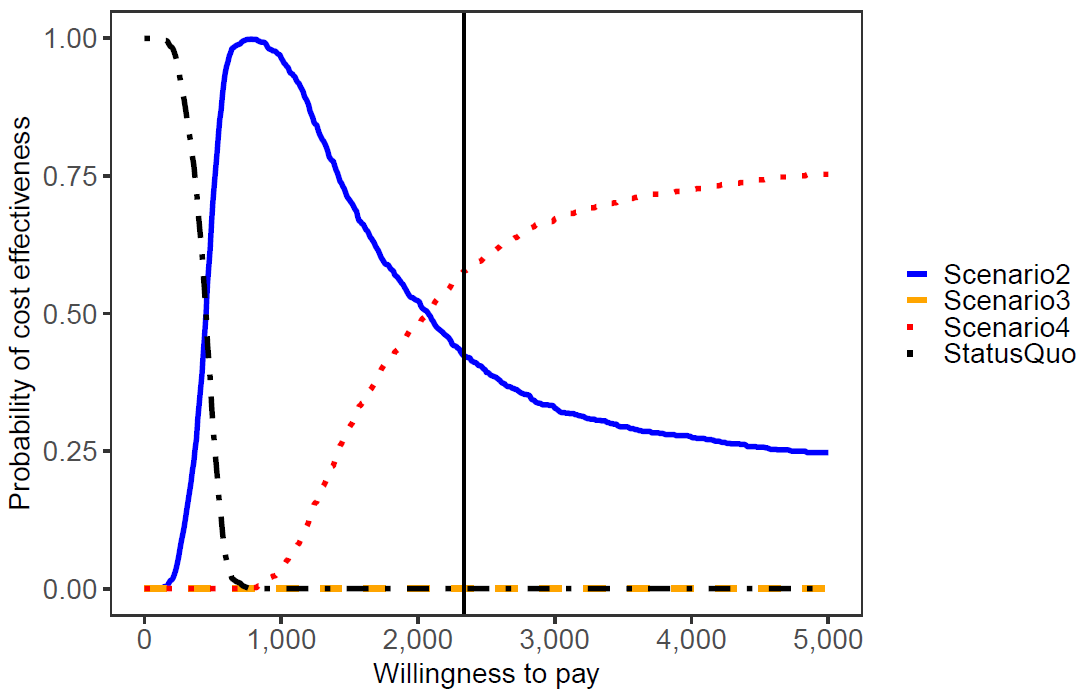
**

**The vertical black line indicates the willingness-to-pay threshold for Vietnam of $2334.**

**Supplementary figure 13:** Expected value of partial perfect information (EVPPI) info-rank plot* for parameter importance comparing scenario 4 (HCV testing and treatment in OAT and ART centres and in the RDS) with the status quo, at a threshold of $2334.

**
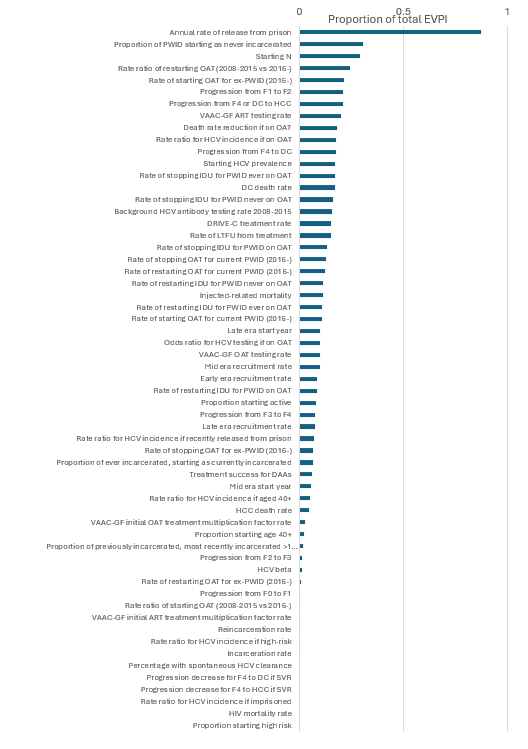
**

*For each parameter and a particular value of the willingness-to-pay threshold, a bar chart is plotted to describe the ratio of EVPPI (specific to that parameter) to EVPI. This represents the relative ‘importance’ of each parameter in terms of the expected value of information.

**Model equations**

The model has 6 levels of compartments: Risk status (r), Age (a), Injecting/OAT status (o), Incarceration Status (p), Liver disease status (d), and HCV infection status (i); see supplementary table 8. The modelled population is given by $Y_{r,a,o,p,i,d}.$

**Supplementary table 9:** Levels of compartments and corresponding indices used in the model equations

| **Compartment level** | **Indices** | **Compartment meaning** |
| --- | --- | --- |
| Risk status | r=1 | Low risk |
|  | r=2 | High risk, no HIV |
|  | r=3 | High risk, HIV |
| Age | a=1 | 16-39 years old |
|  | a=2 | 40+ years old |
| Injecting/OAT status | o=1 | Injector, never on OAT |
|  | o=2 | Injector, currently on OAT |
|  | o=3 | Injector, ever on OAT |
|  | o=4 | Ceased injecting, never on OAT |
|  | o=5 | Ceased injecting, currently on OAT |
|  | o=6 | Ceased injecting, ever on OAT |
| Incarceration status | p=1 | Never incarcerated |
|  | p=2 | Currently incarcerated |
|  | p=3 | Previously incarcerated |
|  | p=4 | Recently released (<12 months) |
| HCV infection status* | i=1 | Never infected (susceptible) |
|  | i=2 | Ab+, RNA- (undiagnosed) |
|  | i=3 | Ab+, RNA+ (undiagnosed) |
|  | i=4 | Ab+, RNA- (Ab status known, RNA unknown) |
|  | i=5 | Ab+, RNA+ (Ab status known, RNA unknown) |
|  | i=6 | Ab+, RNA- (Ab status known, RNA known) |
|  | i=7 | Ab+, RNA+ (Ab status known, RNA known) |
|  | i=8 | Treatment |
|  | i=9 | Previously treated |
|  | i=10 | Ab+, RNA+ (Ab status known, RNA known) in RDS |
| Liver disease | d=1 | F0 |
|  | d=2 | F1 |
|  | d=3 | F2 |
|  | d=4 | F3 |
|  | d=5 | F4 |
|  | d=6 | Decompensated cirrhosis |
|  | d=7 | Hepatocellular carcinoma |

*See supplementary figure 3.

The model can be expressed as a set of ordinary differential equations:

$\frac{dY_{r,a,i,p,i,d}}{dt}=E_{r,a,o,p,i,d}+M_{r,a,o,p,i,d}+ A_{r,a,o,p,i,d}+ O_{r,a,o,p,i,d}+P_{r,a,o,p,i,d}+D_{r,a,o,p,i,d}+I_{r,a,o,p,i,d}+ T_{r,a,o,p,i,d}$

**Entering the Model (**$E_{r,a,o,p,i,d}$)

$E_{r,1,1,p,1,1}=start_{r,1,1,p,1,1}$ for r = 1,2,3 and p = 1,2,3,4

$E_{r,a,o,p,i,d}=0$ otherwise

Where,

$start_{r,1,1,p,1,1}$ is the annual number of people that start injecting with risk ‘r’ and incarceration status ‘p’

**Non-HCV mortality (**$M_{r,a,o,p,i,d}$)

$M_{r,a,o,p,i,d}$ = $-m_{r,a,o}*Y_{r,a,o,p,i,d}$

Where,

$m_{r,a,o}$ is the rate of non-HCV mortality, which differs by risk ‘r’, age ‘a’, and injecting/OAT status ‘o’

**Ageing (**$A_{r,a,o,p,i,d}$)

$A_{r,1,o,p,i,d}= -\epsilon*$ $Y_{r,1,o,p,i,d}$

$A_{r,2,o,p,i,d}= \epsilon*$ $Y_{r,1,o,p,i,d}$

Where,

$\epsilon$ is the rate that people age from 18-39 to 40+.

**Changes in Injecting/OAT status (**$O_{r,a,o,p,i,d}$)

$O_{r,a,1,p,i,d}={\phi_{4}*Y}_{r,a,4,p,i,d}$ - ${(\mu_{1} + Ω_{1} )*Y}_{r,a,1,p,i,d}$

$O_{r,a,2,p,i,d}={\mu_{1} *Y}_{r,a,1,p,i,d}$ + ${ϙ_{3} *Y}_{r,a,3,p,i,d}$+ ${\phi_{5} *Y}_{r,a,5,p,i,d}$- ${(\varpi_{2}+ Ω_{2})*Y}_{r,a,2,p,i,d}$

$O_{r,a,3,p,i,d}$ = ${\varpi_{2} *Y}_{r,a,2,p,i,d}$ + ${\phi_{6} *Y}_{r,a,6,p,i,d}$- ${(ϙ_{3}+ Ω_{3})*Y}_{r,a,3,p,i,d}$

$O_{r,a,4,p,i,d}$ = ${Ω_{1}*Y}_{r,a,1,p,i,d}$ - ${(\mu_{4} + \phi_{4})*Y}_{r,a,4,p,i,d}$

$O_{r,a,5,p,i,d}$ = ${\mu_{4} *Y}_{r,a,4,p,i,d}$ + ${ϙ_{6} *Y}_{r,a,6,p,i,d}$+ ${Ω_{2} *Y}_{r,a,2,p,i,d}$- ${(\varpi_{5}+ \phi_{5})*Y}_{r,a,5,p,i,d}$

$O_{r,a,6,p,i,d}$ = ${\varpi_{5} *Y}_{r,a,5,p,i,d}$ + ${Ω_{3} *Y}_{r,a,3,p,i,d}$- ${(ϙ_{6}+ \phi_{6})*Y}_{r,a,6,p,i,d}$

Where,

$Ω_{o}$is the rate of stopping injecting for people in compartment o

$\phi_{o}$is the rate of restarting injecting for people in compartment o

$\mu_{o}$is the rate of starting OAT for people in compartment o

$\varpi_{o}$is the rate of stopping OAT for people in compartment o

$ϙ_{o}$is the rate of restarting OAT for people in compartment o

**Incarceration (**$P_{r,a,o,p,i,d}$)

$P_{r,a,o,1,i,d}$ = ${-\psi*Y}_{r,a,o,1,i,d}$

$P_{r,a,o,2,i,d}=0$ for 0=2,5 or i = 8,10

$P_{r,a,1,2,i,d}$ = ${\psi*Y}_{r,a,1,1,i,d}$ + ${\lambda*Y}_{r,a,1,3,i,d}$ + ${\lambda*Y}_{r,a,1,4,i,d}$ - ${\delta*Y}_{r,a,1,2,i,d}$ for i=1,2,3,4,5,6,9

$P_{r,a,1,2,7,d}=\sum_{i=7,8,10} \left( {\psi*Y}_{r,a,1,1,i,d} + {\lambda*Y}_{r,a,1,3,i,d} + {\lambda*Y}_{r,a,1,4,i,d} \right)- {\delta*Y}_{r,a,1,2,7,d}$

$P_{r,a,3,2,i,d}$ = $\sum_{o=2,3} \left( {\psi*Y}_{r,a,o,1,i,d} + {\lambda*Y}_{r,a,o,3,i,d} + {\lambda*Y}_{r,a,o,4,i,d} \right)- {\delta*Y}_{r,a,3,2,i,d}$ for i=1,2,3,4,5,6,9

$P_{r,a,3,2,7,d}=\sum_{o=2,3} \sum_{i=7,8,10} ({\psi*Y}_{r,a,o,1,i,d} + {\lambda*Y}_{r,a,o,3,i,d} + {\lambda*Y}_{r,a,1,o,i,d} ) - {\delta*Y}_{r,a,3,2,7,d}$

$P_{r,a,4,2,i,d}$ = ${\psi*Y}_{r,a,4,1,i,d}$ + ${\lambda*Y}_{r,a,4,3,i,d}$ + ${\lambda*Y}_{r,a,4,4,i,d}$ - ${\delta*Y}_{r,a,4,2,i,d}$ for i=1,2,3,4,5,6,9

$P_{r,a,4,2,7,d}=\sum_{i=7,8,10} \left( {\psi*Y}_{r,a,4,1,i,d} + {\lambda*Y}_{r,a,4,3,i,d} + {\lambda*Y}_{r,4,1,4,i,d} \right)- {\delta*Y}_{r,a,4,2,7,d}$

$P_{r,a,6,2,i,d}$ = $\sum_{o=5,6} \left( {\psi*Y}_{r,a,o,1,i,d} + {\lambda*Y}_{r,a,o,3,i,d} + {\lambda*Y}_{r,a,o,4,i,d} \right)- {\delta*Y}_{r,a,6,2,i,d}$ for i=1,2,3,4,5,6,9

$P_{r,a,6,2,7,d}=\sum_{o=5,6} \sum_{i=7,8,10} ({\psi*Y}_{r,a,o,1,i,d} + {\lambda*Y}_{r,a,o,3,i,d} + {\lambda*Y}_{r,a,o,4,i,d} ) - {\delta*Y}_{r,a,6,2,7,d}$

$P_{r,a,o,2,i,d}$ = ${\psi*Y}_{r,a,o,1,i,d}$ + ${\lambda*Y}_{r,a,o,3,i,d}$ + ${\lambda*Y}_{r,a,o,4,i,d}$ - ${\delta*Y}_{r,a,o,2,i,d}$

$P_{r,a,o,3,i,d}$ = ${\pi*Y}_{r,a,o,4,i,d}$ - ${\lambda*Y}_{r,a,io,3,i,d}$

$P_{r,a,o,4,i,d}$ = ${\delta*Y}_{r,a,o,2,i,d}$ - ${(\pi+ \lambda)*Y}_{r,a,o,4,i,d}$

Where,

$\psi$ is the rate of incarceration

$\delta$ is the rate of release

$\lambda$ is the rate of re-incarceration

$\pi$ is the rate of transitioning out of a high-risk post-release period

**HCV transmission (**$I_{r,a,o,p,i,d}$)

$I_{r,a,o,p,1,d}$ = -${Inf}_{r,a,o,p}$ ${*Y}_{r,a,o,p,1,d}$

$I_{r,a,o,p,2,d}$ =${Clear*Inf}_{r,a,o,p}{*Y}_{r,a,o,p,1,d}$ - $\left( 1-Clear \right)*{{Inf}_{r,a,o,p}*Y}_{r,a,o,p,2,d}$

$I_{r,a,o,p,3,d}$ =$(1-{Clear)*Inf}_{r,a,o,p}{*(Y}_{r,a,o,p,1,d}$ +$Y_{r,a,o,p,2,d} )$

$I_{r,a,o,p,4,d}$ = - $\left( 1-Clear \right)*{{Inf}_{r,a,o,p}*Y}_{r,a,o,p,4,d}$

$I_{r,a,o,p,5,d}$ =$\left( 1-Clear \right)*{Inf}_{r,a,o,p}*(Y_{r,a,o,p,4,d}+Y_{r,a,o,p,6,d}+Y_{r,a,o,p,9,d})$

$I_{r,a,o,p,6,d}$ =- $\left( 1-Clear \right)*{Inf}_{r,a,o,p}$ ${*Y}_{r,a,o,p,6,d}$

$I_{r,a,o,p,9,d}$ = - $\left( 1-Clear \right)*Reinf*{{Inf}_{r,a,o,p}*Y}_{r,a,o,p,9,d}$

$I_{r,a,o,p,i,d}=0$ for i=7,8,10

Where,

${Inf}_{r,a,o,p}$ is the rate in which people acquire HCV, which differs by risk ‘r’, age ‘a’, injecting/OAT status ‘o’, and incarceration ‘p’. This is described in the “Force of Infection” section below

$Clear$ is the proportion of people acquiring HCV who immediately spontaneously clear their infection

$Reinf$ is the relative ratio of reinfection following treatment compared with primary infection

**Force of infection (**${Inf}_{r,a,o,p}$**)**

${Inf}_{r,a,o,p}=\eta_{r,a,o,p}*\beta*\frac{\sum_{r} \sum_{a} \sum_{o=1,2,3} \sum_{p=1,3,4} \sum_{i=3,5,7,10} \sum_{d} \eta_{r,a,o,p}*Y_{r,a,o,p,i,d}}{\sum_{r} \sum_{a} \sum_{o=1,2,3} \sum_{p=1,3,4} \sum_{i} \sum_{d} \eta_{r,a,o,p}*Y_{r,a,o,p,i,d}}$ for $o\leq3$and $p\neq2$

${Inf}_{r,a,o,2}=\eta_{r,a,o,2}*\beta*\frac{\sum_{r} \sum_{a} \sum_{o=1,2,3} \sum_{i=3,5,7,10} \sum_{d} Y_{r,a,o,2,i,d}}{\sum_{r} \sum_{a} \sum_{o=1,2,3} \sum_{i} \sum_{d} Y_{r,a,o,2,i,d}}$ for $o\leq3$and $p=2$

${Inf}_{r,a,o,p}=0$ for $o\geq4$

Where,

$\beta$ is the HCV transmission rate

$\eta_{r,a,o,p}$is the HCV injecting transmission risk of PWID relative to PWID who are low risk (r=1), aged 18-39 years old (a=1), never on OAT (o=1), and never incarcerated (p=1) . By definition, $\eta_{1,1,1,1}=1$.

**HCV diagnosis and treatment** ($T_{r,a,o,p,i,d}$)

$T_{r,a,o,p,1,d}$ = 0

$T_{r,a,o,2,i,d}$ = 0

$T_{r,a,o,p,2,d}$ = - ${(Abdiag}_{o}{+AbRNAdiag}_{r,o}$ ${)*Y}_{r,a,o,p,2,d}$ for p$\neq2$

$T_{r,a,o,p,3,d}$ =$-{({RDSdiag}_{o}+Abdiag}_{o,}{+AbRNAdiagr,}_{o}$ ${)*Y}_{r,a,o,p,3,d}$ for p$\neq2$

$T_{r,a,o,p,4,d}$ =${Abdiag}_{o,}{*Y}_{r,a,o,p,2,d}$ - ${RNAdiag}_{o}$ ${*Y}_{r,a,o,p,4,d}$ for p$\neq2$

$T_{r,a,o,p,5,d}$ =${Abdiag}_{o}{*Y}_{r,a,o,p,3,d}$- ${({RDSdiag}_{o,}+RNAdiag}_{o}$ ${)*Y}_{r,a,o,p,5,d} for p\neq2$

$T_{r,a,o,p,6,d}$=${RNAdiag}_{o,}{*Y}_{r,a,o,p,4,d}$ + ${AbRNAdiag}_{o}{*Y}_{r,a,o,p,2,d}$ for p$\neq2$

$T_{r,a,o,p,7,d}$=${AbRNAdiag}_{r,o}{*Y}_{r,a,o,p,3,d}$ + ${RNAdiag}_{o}{*Y}_{r,a,o,p,5,d}$ + ${LeaveRDS}_{o}{*Y}_{r,a,o,p,10,d}$ + ${Fail*Y}_{r,a,o,p,8,d}$ - ${({RDSdiag}_{o}+Treat}_{r,o}$ ${)*Y}_{r,a,o,p,7,d}$ for p$\neq2$

$T_{r,a,o,p,8,d}$ =${Treat}_{r,o}{*Y}_{r,a,o,p,7,d}$ + ${TreatRDS}_{o}{*Y}_{r,a,o,p,10,d}$ - $(Fail+Success{)*Y}_{r,a,o,p,8,d}$ for p$\neq2$

$T_{r,a,o,p,9,d}$=$Success{*Y}_{r,a,o,p,8,d}$ for p$\neq2$

$T_{r,a,o,p,10,d}$=${RDSdiag}_{o}{*Y}_{r,a,o,p,3,d}$ + ${RDSdiag}_{o}{*Y}_{r,a,o,p,5,d}$ + ${RDSdiag}_{o}{*Y}_{r,a,o,p,7,d}$ - ${({TreatRDS}_{o}+LeaveRDS}_{o}{)*Y}_{r,a,o,p,10,d}$ for p$\neq2$

Where,

${Abdiag}_{o}$ is the rate of HCV antibody diagnosis, which differs by injecting/OAT status ‘o’

${RNAdiag}_{o}$ is the rate of HCV RNA diagnosis, which differs by injecting/OAT status ‘o’

${AbRNAdiag}_{r,o}$ is the rate of HCV combined Ab and RNA diagnosis, which differs by injecting/OAT status ‘o’ and risk status ‘r’

${Treat}_{r,o}$ is the rate of HCV treatment, which differs by injecting/OAT status ‘o’ and risk status ‘r’

${RDSdiag}_{r,o}$ is the rate of HCV Ab and RNA diagnosis during an RDS surve, which differs by injecting/OAT status ‘o’

${TreatRDS}_{o}$ is the rate of starting HCV treatment among people participating in RDS surveys, which differs by injecting/OAT status ‘o’

$Success$is the rate of successful HCV treatment

$Fail$is the rate of unsuccessful HCV treatment

$LeaveRDS$is the rate that people in the RDS who are HCV Ab+ RNA+ who were not treated leave the RDS

**Liver disease status (**$D_{r,a,o,p,i,d}$)

$D_{r,a,o,p,i,1}=0$ for i=1,2,4,6,8

$D_{r,a,o,p,i,1}$ = ${-f^{1}*Y}_{r,a,o,p,i,1}$ for i=3,5,7,10

$D_{r,a,o,p,i,2}=0$ for i=1,2,4,6,8

$D_{r,a,o,p,i,2}$ = ${f^{1}*Y}_{r,a,o,p,i,1}{-f^{2}*Y}_{r,a,o,p,i,2}$ for i=3,5,7,10

$D_{r,a,o,p,i,3}=0$ for i=1,2,4,6,8

$D_{r,a,o,p,i,3}$ = ${f^{2}*Y}_{r,a,o,p,i,2}$ ${-f^{3}*Y}_{r,a,o,p,i,3}$ for i=3,5,7,10

$D_{r,a,o,p,i,4}=0$ for i=1,2,4,6,8

$D_{r,a,o,p,i,4}$ = ${f^{3}*Y}_{r,a,o,p,i,3}$ $-{f^{4}*Y}_{r,a,o,p,i,4}$ for i=3,5,7,10

$D_{r,a,o,p,i,5}$ = $-{(f_{i}^{5}+f_{i}^{6})*Y}_{r,a,o,p,i,5}$ for i=1,2,4,6,8

$D_{r,a,o,p,i,5}$ = ${f^{4}*Y}_{r,a,o,p,i,4}$ $-{(f_{i}^{5}+f_{i}^{6})*Y}_{r,a,o,p,i,5}$ for i=3,5,7,10

$D_{r,a,o,p,i,6}$ = ${f_{i}^{5}*Y}_{r,a,o,p,i,5}$ ${-f_{i}^{7}*Y}_{r,a,o,p,i,6}$ $-{DCm*Y}_{r,a,o,p,i,6}$

$D_{r,a,o,p,i,7}$ = ${f_{i}^{6}*Y}_{r,a,o,p,i,5}$+ ${f_{i}^{7}*Y}_{r,a,o,p,i,6}$ ${-HCCm*Y}_{r,a,o,p,i,7}$

Where,

$f^{1}$ is the rate of progressing from F0 to F1 for HCV RNA+ people

$f^{2}$ is the rate of progressing from F1 to F2 for HCV RNA+ people

$f^{3}$ is the rate of progressing from F2 to F3 for HCV RNA+ people

$f^{4}$ is the rate of progressing from F3 to F4 for HCV RNA+ people

$f_{i}^{5}$ is the rate of progressing from F4 to DC, which differs by infection status ‘i’

$f_{i}^{6}$is the rate of progressing from F4 to HCC, which differs by infection status ‘i’

$f_{i}^{7}$ is the rate of progressing from DC to HCC, which differs by infection status ‘i’

$DCm$ is the rate of decompensated cirrhosis-related mortality

$HCCm$ is the rate of hepatocellular carcinoma cirrhosis-related mortality

(Note that we are assuming there is no progression whilst on treatment)

The model code will be made available upon request to the corresponding author.

**References**

1. Moles JP, Vallo R, Khue PM, Huong DT, Oanh KTH, Thoa NT, et al. HIV control programs reduce HIV incidence but not HCV incidence among people who inject drugs in HaiPhong, Vietnam. Sci Rep. 2020;10(1):6999.

2. Des Jarlais DC, Arasteh K, Huong DT, Oanh KTH, Feelemyer JP, Khue PM, et al. Using large-scale respondent driven sampling to monitor the end of an HIV epidemic among persons who inject drugs in Hai Phong, Viet Nam. Plos One. 2021;16(11).

3. Des Jarlais DC, Huong DT, Oanh KTH, Feelemyer JP, Arasteh K, Khue PM, et al. Ending an HIV epidemic among persons who inject drugs in a middle-income country: extremely low HIV incidence among persons who inject drugs in Hai Phong, Viet Nam. Aids. 2020;34(15):2305-11.

4. Des Jarlais DC, Huong DT, Oanh KTH, Pham MK, Giang HT, Thanh NTT, et al. Prospects for ending the HIV epidemic among persons who inject drugs in Haiphong, Vietnam. Int J Drug Policy. 2016;32:50-6.

5. World Population Prospects 2019, Online Edition [Internet]. 2019.

6. Degenhardt L, Bucello C, Mathers B, Briegleb C, Ali H, Hickman M, McLaren J. Mortality among regular or dependent users of heroin and other opioids: a systematic review and meta-analysis of cohort studies. Addiction. 2011;106(1):32-51.

7. Stone J, Fraser H, Lim AG, Walker JG, Ward Z, MacGregor L, et al. Incarceration history and risk of HIV and hepatitis C virus acquisition among people who inject drugs: a systematic review and meta-analysis. Lancet Infect Dis. 2018;18(12):1397-409.

8. Manns MP, Maasoumy B. Breakthroughs in hepatitis C research: from discovery to cure. Nat Rev Gastro Hepat. 2022;19(8):533-50.

9. Nagot N, Binh NT, Hong TT, Vinh VH, Quillet C, Vallo R, et al. A community-based strategy to eliminate hepatitis C among people who inject drugs in Vietnam. Lancet Reg Health West Pac. 2023;37:100801.

10. Des Jarlais D, Khue PM, Feelemyer J, Arasteh K, Huong DT, Oanh KTH, et al. Using dual capture/recapture studies to estimate the population size of persons who inject drugs (PWID) in the city of Hai Phong, Vietnam. Drug Alcohol Depen. 2018;185:106-11.

11. Platt L, Minozzi S, Reed J, Vickerman P, Hagan H, French C, et al. Needle syringe programmes and opioid substitution therapy for preventing hepatitis C transmission in people who inject drugs. Cochrane Db Syst Rev. 2017(9).

12. Smith DJ, Combellick J, Jordan AE, Hagan H. Hepatitis C virus (HCV) disease progression in people who inject drugs (PWID): A systematic review and meta-analysis. Int J Drug Policy. 2015;26(10):911-21.

13. Shepherd J, Jones J, Hartwell D, Davidson P, Price A, Waugh N. Interferon alpha (pegylated and non-pegylated) and ribavirin for the treatment of mild chronic hepatitis C: a systematic review and economic evaluation. Health Technol Assess. 2007;11(11):1-205, iii.

14. Hallager S, Ladelund S, Christensen PB, Kjær M, Roege BT, Gronbæk KE, et al. Liver-related morbidity and mortality in patients with chronic hepatitis C and cirrhosis with and without sustained virologic response. Clin Epidemiol. 2017;9:501-16.

15. Morgan RL, Baack B, Smith BD, Yartel A, Pitasi M, Falck-Ytter Y. Eradication of Hepatitis C Virus Infection and the Development of Hepatocellular Carcinoma A Meta-analysis of Observational Studies. Ann Intern Med. 2013;158(5):329-+.

16. Vinh VH, Vallo R, Giang HT, Huong DT, Oanh KTH, Khue PM, et al. A cohort study revealed high mortality among people who inject drugs in Hai Phong, Vietnam. J Clin Epidemiol. 2021;139:38-48.

17. Degenhardt L, Grebely J, Stone J, Hickman M, Vickerman P, Marshall BDL, et al. Global patterns of opioid use and dependence: harms to populations, interventions, and future action. Lancet. 2019;394(10208):1560-79.

18. Mathers BM, Degenhardt L, Bucello C, Lemon J, Wiessing L, Hickman M. Mortality among people who inject drugs: a systematic review and meta-analysis. B World Health Organ. 2013;91(2):102-23.

19. International Monetary Fund. GDP per capita, current prices 2023 [Available from: <https://www.imf.org/external/datamapper/NGDPDPC@WEO/THA/IDN/PHL/VNM/MYS>.

20. Ochalek J, Lomas J, Claxton K. Estimating health opportunity costs in low-income and middle-income countries: a novel approach and evidence from cross-country data (vol 3, e000964, 2018). Bmj Glob Health. 2019;4(3).

21. Salomon JA, Haagsma JA, Davis A, de Noordhout CM, Polinder S, Havelaar AH, et al. Disability weights for the Global Burden of Disease 2013 study. Lancet Glob Health. 2015;3(11):E712-E23.

22. Diseases GBD, Injuries C. Global incidence, prevalence, years lived with disability (YLDs), disability-adjusted life-years (DALYs), and healthy life expectancy (HALE) for 371 diseases and injuries in 204 countries and territories and 811 subnational locations, 1990-2021: a systematic analysis for the Global Burden of Disease Study 2021. Lancet. 2024;403(10440):2133-61.

23. Walker JG, Mafirakureva N, Iwamoto M, Campbell L, San Kim C, Hastings RA, et al. Cost and cost-effectiveness of a simplified treatment model with direct-acting antivirals for chronic hepatitis C in Cambodia. Liver Int. 2020;40(10):2356-66.

24. Baio G, Berardi A, Heath A. Bayesian Cost Effectiveness Analysis with the R package BCEA. New York, NY.: Springer; 2017.

25. Dennis BB, Naji L, Jajarmi Y, Ahmed A, Kim D. New hope for hepatitis C virus: Summary of global epidemiologic changes and novel innovations over 20 years. World J Gastroentero. 2021;27(29):4818-30.
